# Identification of key molecular targets for stachyose in hepatocellular carcinoma: focus on STAT3 and FN1

**DOI:** 10.3389/fonc.2025.1576449

**Published:** 2025-07-23

**Authors:** Haihua Chen, Xianyou Wang, Jiongcheng Ying, YuQin Huang, Yuxi Liu, Yifu Feng, Binbo Fang

**Affiliations:** ^1^ Department of Hepatobiliary Surgery, Taizhou Hospital of Zhejiang Province affiliated to Wenzhou Medical University, Linhai, Zhejiang, China; ^2^ Department of Surgery, Taizhou Hospital of Zhejiang Province affiliated to Wenzhou Medical University, Linhai, Zhejiang, China; ^3^ Department of Medicine, Taizhou University, Jiaojiang, China; ^4^ Department of Hepatobiliary Surgery, Taizhou Central Hospital (an affiliated Hospital of Taizhou University), Jiaojiang, China

**Keywords:** stachyose, hepatocellular carcinoma, STAT3, FN1, JAK

## Abstract

**Background and objective:**

Hepatocellular carcinoma (HCC) ranks among the most prevalent malignancies on a global scale. Stachyose (STA), an oligosaccharide widely present in legumes, has demonstrated various biological activities, including improving gut microbiota, anti-oxidative stress, and anti-tumor proliferation. This study aimed to predict potential targets of STA in HCC treatment and to identify key hub genes.

**Methods:**

By integrating multiple public databases and bioinformatics tools, we screened 34 candidate targets and constructed STA’s action network and PPI network in HCC. Functional enrichment analysis revealed 10 relevant KEGG pathways and key features related to cellular components, molecular functions, and biological processes. Finally, we conducted molecular docking, single gene analysis, and *in vitro* experimental validation on core targets.

**Results:**

Through screening of multiple databases, we identified 34 common targets associated with STA and HCC and subsequently constructed a protein-protein interaction (PPI) network. Through this analysis, we ultimately selected STAT3 and FN1 as core hub genes. Functional and pathway analyses indicated that these targets participate in multiple cancer-related pathways and have significant roles in cellular components, biological processes, and molecular functions. The results indicated a positive correlation between the expression of STAT3 and FN1 with angiogenesis, tumor inflammation, and epithelial-mesenchymal transition (EMT). Molecular docking experiments validated the stable binding capacity between STA and these core genes, and *in vitro* experiments further confirmed that STA could inhibit HCC cell proliferation and migration by downregulating STAT3 and FN1 expression.

**Conclusion:**

This study offers a comprehensive exploration of the molecular mechanisms through which STA may treat HCC, identifying STAT3 and FN1 as key targets and validating their clinical relevance and potential for application.

## Introduction

Hepatocellular carcinoma (HCC) represents one of the most prevalent malignancies globally, constituting over 90% of primary liver cancer cases (1). In recent decades, both the incidence and mortality rates of HCC have markedly increased, especially in regions with high prevalence, such as East Asia and sub-Saharan Africa. Principal risk factors encompass chronic infections with hepatitis B virus (HBV) and hepatitis C virus (HCV), alcoholic liver disease, non-alcoholic fatty liver disease (NAFLD), and aflatoxin exposure (2, 3). However, due to the absence of clear early symptoms, many patients are diagnosed at advanced stages, leading to a poor prognosis with a five-year survival rate below 20%. Traditional treatment modalities, encompassing surgical resection, radiotherapy, and chemotherapy, exhibit limited efficacy against hepatocellular carcinoma (HCC) and are frequently accompanied by drug resistance and substantial adverse effects. Therefore, the development of novel therapeutic strategies is imperative. In recent years, the advent of targeted therapy and immunotherapy has offered new hope for HCC treatment (4, 5). Targeted therapy works by identifying and inhibiting tumor cell-specific molecular targets, thereby minimizing damage to normal cells and enhancing treatment selectivity and efficacy (6).

Stachyose (STA) is an oligosaccharide, a type of low molecular weight sugar commonly found in various plants, especially legumes such as soybeans and peas, as well as some root vegetables (7). It helps inhibit tumor cell growth by promoting the proliferation of beneficial gut microbiota, improving the intestinal microenvironment, and enhancing the body’s immune function (8). Additionally, STA possesses antioxidant properties that scavenge free radicals and reduce oxidative stress-induced cellular damage, thereby inhibiting the proliferation of certain tumor cell types (9–11). In a study, Stachydrine (an alkaloid related to Stachyose) was found to inhibit epithelial-mesenchymal transition (EMT) induced by transforming growth factor-β1 (TGF-β1), a process closely associated with tumor invasion and metastasis (12). Research demonstrated that Stachydrine significantly reduced the migration and invasion capabilities of HepG2 liver cancer cells by suppressing the activation of TGF-β/Smad and PI3K/Akt/mTOR signaling pathways. This finding suggests that Stachyose might exert its anti-tumor effects through similar mechanisms. Additionally, Stachyose may influence tumor cell survival and death by regulating intracellular redox balance. Similar to Stachydrine, another compound, Solasonine, was discovered to promote ferroptosis in liver cancer cells by disrupting the glutathione redox system (13). The discovery of this mechanism provides new insights into the potential application of Stachyose in HCC treatment. In conclusion, research on Stachyose and its related compounds in HCC demonstrates their potential as prospective therapeutic agents.

This study aims to systematically predict and validate potential targets of STA and elucidate its molecular mechanisms in HCC treatment using comprehensive bioinformatics methods. First, we utilized the Swiss Target Prediction database to identify potential targets of STA, which were then integrated and deduplicated through GeneCards, OMIM, the Therapeutic Target Database, and PHARMGKB to select those relevant to HCC. Utilizing Venn diagram analysis, we identified shared targets between STA and HCC and subsequently constructed a protein-protein interaction (PPI) network using the STRING database to identify hub genes. To evaluate the clinical significance of these hub genes, we performed single-gene expression and prognostic analyses, investigating their associations with key tumor biological processes, including angiogenesis, tumor inflammation, tumor proliferation, and epithelial-mesenchymal transition (EMT). Molecular docking experiments validated the binding capacity of STA with the hub genes, while *in vitro* experiments were conducted to confirm STA’s regulatory effects on these genes. This study not only provides a theoretical foundation for considering STA as a targeted therapeutic agent for HCC but also highlights the potential application value of STAT3 and FN1 in HCC treatment, offering new insights and directions for targeted therapy research in this context.

## Materials and methods

### Materials

Stachyose was purchased from MedChemExpress. Primary antibodies for anti-p-STAT3 (AP0705), anti-p-JAK2 (AP0531), anti-Fibronectin (A12977), and anti-β-actin (AC026) were obtained from ABclonal. PVDF membranes (IPFL00010) were sourced from Millipore (USA), and fetal bovine serum (10099-141) was acquired from Gibco (USA).

### Target prediction

We collected 100 potential targets of STA using the Swiss Target Prediction database. These targets were subsequently categorized and analyzed based on their functions. To identify HCC-related targets, we collected relevant targets from GeneCards database (Relevance score ≥10), OMIM database, Therapeutic Target Database, and PHARMGKB database. After merging and deduplication, a total of 2434 targets were obtained. Using Venn diagram analysis, 34 common targets between STA and liver cancer were identified.

### PPI network construction and hub gene identification

The PPI network of the 34 key targets was constructed utilizing the STRING database (Search Tool for the Retrieval of Interacting Genes/Proteins). Subsequently, the PPI network was imported into Cytoscape version 3.9.1 for visualization and analysis. Node topology parameters were calculated using the CytoNCA plugin within Cytoscape. Genes were scored based on Betweenness, Closeness, Degree, Eigenvector, LAC, and Network, retaining genes above their median values. After two rounds of screening, 3 hub genes were identified.

### KEGG and GO enrichment analysis

For gene set functional enrichment analysis, we utilized the KEGG REST API (https://www.kegg.jp/kegg/rest/keggapi.html) to acquire the latest KEGG Pathway gene annotations. After mapping our genes to this background, we performed enrichment analysis using the R package clusterProfiler (version 3.14.3), setting a minimum gene set size of 5, a maximum of 5000, and defining statistical significance as P-value <0.05 and FDR <0.25. For GO annotation, we used the R package org.Hs.eg.db (version 3.1.0) as the background. The enrichment analysis was conducted with the same parameters as the KEGG analysis.

### Single gene analysis

RNAseq data and clinical information for liver cancer were sourced from The Cancer Genome Atlas (TCGA) (https://portal.gdc.com). Statistical analysis was conducted using R v4.0.3, primarily employing the rank-sum test for group comparisons, with P-values <0.05 deemed significant.

### Prognostic analysis

RNAseq data and clinical information for liver cancer were obtained from The TCGA database (https://portal.gdc.com). We used the log-rank test to assess survival differences in Kaplan-Meier (KM) analysis and conducted timeROC analysis to evaluate gene predictive accuracy. P-values and hazard ratios (HR) with 95% confidence intervals (CI) for KM curves were derived from log-rank tests and univariate Cox regression. All analyses were performed using R software v4.0.3 (R Foundation for Statistical Computing, 2020), with P < 0.05 considered statistically significant.

### Gene-pathway association

RNAseq data and clinical information for liver cancer tumors were sourced from The TCGA database (https://portal.gdc.com). Genes in relevant pathways were analyzed using the GSVA package in R software v4.0.3 with the ‘ssgsea’ method. Spearman correlation analysis was then conducted between genes and pathway scores. A p-value of less than 0.05 was deemed statistically significant.

### Cell immunofluorescence experiment

First, culture hepatocellular carcinoma cells to an appropriate density and treat them with the suitable culture medium. Then, fix the cells using 4% paraformaldehyde for about 30 minutes. After fixation, wash the cells with PBS buffer to remove the fixative. Next, permeabilize the cells using PBS solution containing 0.1% Triton X-100 for 15 minutes to increase antibody penetration. After permeabilization, block non-specific binding by incubating the cells with 5% BSA (bovine serum albumin) for 1 hour. Then, add the primary antibody and incubate overnight at 4°C. After incubation, wash the cells with PBS to remove any unbound antibodies, then add the secondary antibody and incubate at room temperature for 1 hour. After the second incubation, wash the cells with PBS again to remove excess secondary antibody. Finally, stain the cell nuclei with DAPI for 5–10 minutes. Once completed, observe the cells under a fluorescence microscope and capture images.

### Molecular docking

Molecular docking was conducted using AutoDock 1.5.6 software (http://vina.scripps.edu/). Initially, Pymol 2.1.0 was employed to prepare the necessary ligands and proteins. Crystal structures for target proteins were sourced from the PDB database (https://www.rcsb.org/) and underwent preprocessing, which included removing excess hydrogen atoms, modifying amino acids, optimizing energy, and adjusting force field parameters. AutoDock Tools 1.5.6 (http://vina.scripps.edu/) was then used to add hydrogen atoms and assign charges to the proteins, saving them in pdbqt format. Ligand structures were obtained from the PubChem database (https://pubchem.ncbi.nlm.nih.gov/). Finally, molecular docking was performed using the vina tool in PyRx software (https://pyrx.sourceforge.io/), with the Affinity (kcal/mol) value reflecting binding strength; lower values indicate more stable ligand-receptor interactions. Finally, 3D images of docking results were visualized using Pymol, while 2D images were analyzed using Discovery Studio 2019.

### Cell culture

HepG2 cells (CL-0103) were procured from Wuhan Procell Life Science & Technology Co., Ltd. The cells were cultured in high-glucose Dulbecco’s Modified Eagle Medium (DMEM) supplemented with 10% fetal bovine serum and 1% penicillin-streptomycin. Subsequently, the culture medium was treated with STA at a concentration of 1.6 mg/mL.

### Clonogenic assay

Cells were grown to the logarithmic phase, collected, counted, and diluted before seeding into culture dishes. The dishes were incubated at 37°C with 5% CO_2_ for 10–14 days until visible clones appeared. Then, cell clones were fixed with methanol or ethanol and stained with dye for easy identification. Finally, they were observed under a microscope.

### Transwell migration assay

First, culture medium supplemented with 10% FBS was added to the lower chamber for the migration assay. Liver cancer cells were collected and adjusted to a concentration of 1×10^6^ cells/mL, then resuspended in the culture medium. A 200 µL aliquot of this suspension was placed in the upper chamber, which was incubated in a CO_2_ incubator for 48 hours. After incubation, non-migrated cells in the upper chamber were removed using sterile cotton swabs, and the migrated cells in the lower chamber were counted. Data were recorded for comparison and statistical analysis. Sterile operations were ensured to avoid cross-contamination.

### Western blot experiment

Cell samples were collected, and total protein was extracted with lysis buffer. Protein concentration was measured using the BCA method. Equal protein amounts were then mixed with loading buffer and heated to denature. Next, samples were loaded onto polyacrylamide gels for electrophoretic separation. After electrophoresis, proteins were transferred to membranes (PVDF), which were blocked to reduce non-specific binding. Subsequently, specific primary antibodies targeting the proteins of interest were added, followed by incubation and washing. Secondary antibodies were then added and incubated. Finally, protein signals were detected by chemiluminescence and recorded using an imaging system.

### Statistical analysis

Statistical analyses and graphing were performed using GraphPad Prism 8.0.2. Normally distributed data are presented as mean ± standard deviation. Differences between two independent groups were assessed using the t-test, with p-values below 0.05 considered statistically significant.

## Results

### Prediction of STA targets for HCC treatment and construction of PPI network

**Figure 1:**
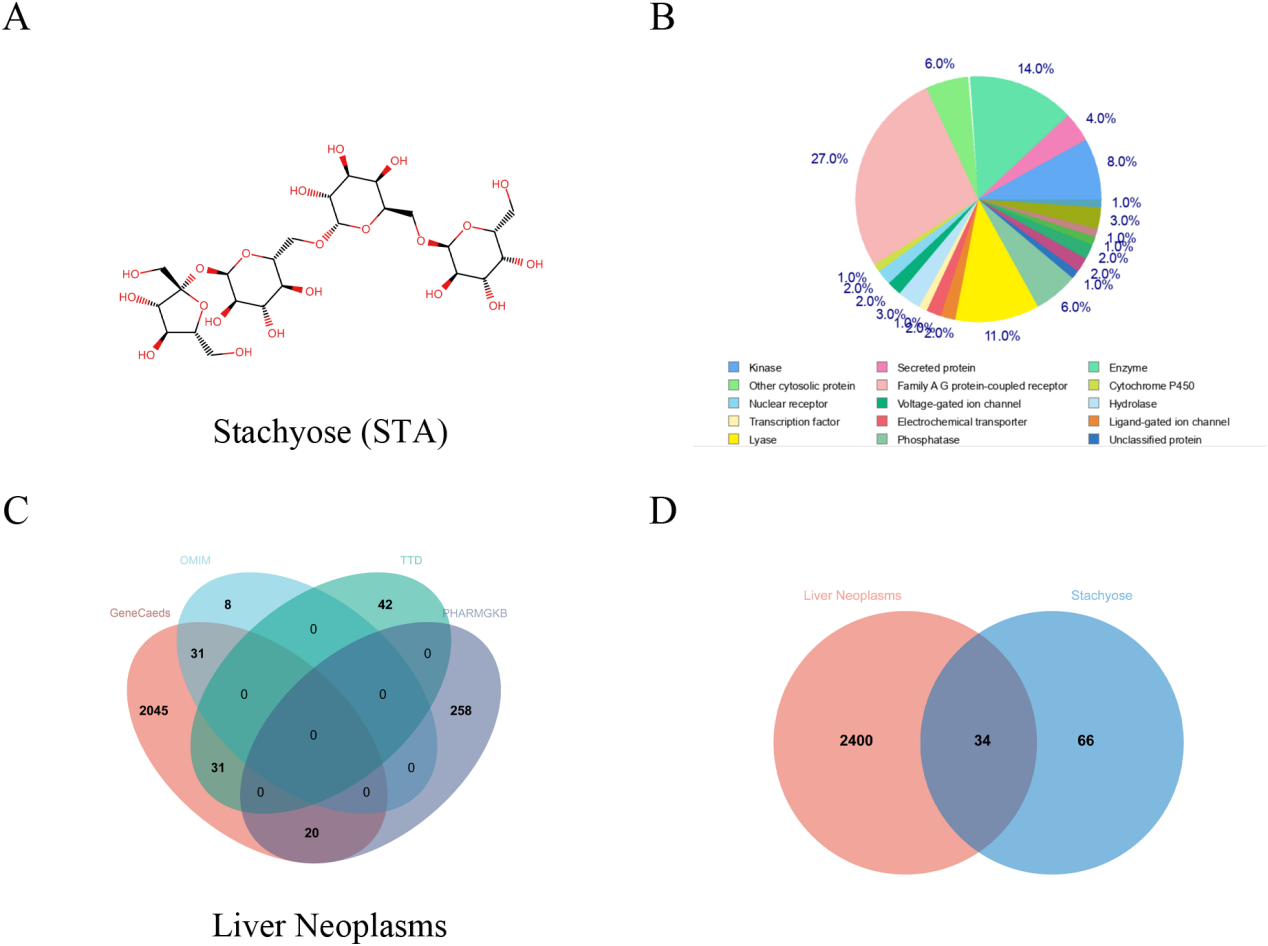
Common genes between STA and HCC. **(A)** Chemical structure of STA. **(B)** Classification of STA targets. **(C)** Venn diagram of HCC-related genes from various databases. **(D)** Venn diagram of STA and HCC.

**Figure 2:**
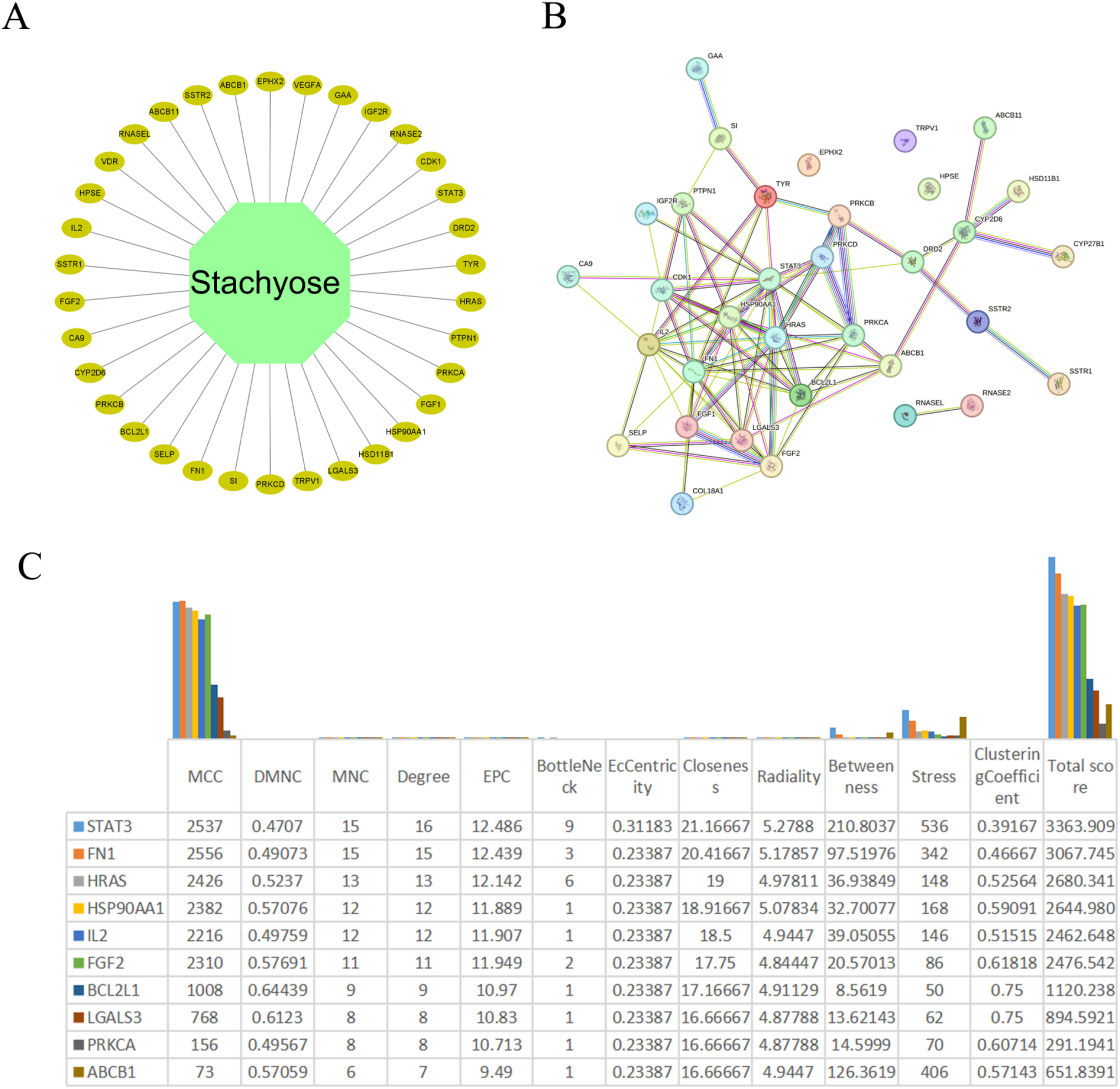
Identification of hub genes. **(A)** Network diagram of cross-genes between STA and HCC. **(B)** PPI network of common genes. **(C)** The scores of various indices for the top ten genes after processing with cytohubba.

Using the Swiss Target Prediction database, we identified 100 potential targets of stachyose (STA) and analyzed them based on function (**Figures 1A, B**). After merging and deduplicating data from GeneCards (with a relevance score ≥10), OMIM, the Therapeutic Target Database, and PHARMGKB, we collected a total of 2,434 targets related to HCC (**Figure 1C**). A Venn diagram revealed 34 common targets between STA and liver cancer (**Figures 1D**, **2A**). A PPI network for the 34 identified key targets was constructed utilizing the STRING database, as illustrated in **Figure 2B**.

### Hub genes for STA treatment of liver cancer

The PPI network was imported into Cytoscape version 3.9.1 for visualization and analysis. Subsequently, node topology parameters were computed utilizing the CytoNCA plugin. Subsequently, we processed the PPI data using the cytohubba plugin and presented the top ten ranked genes (**Figure 2C**). In **Figure 2C**, we found that FN1 and STAT3 had the highest scores; therefore, we selected these two genes as core genes for subsequent related analyses.

### GO and KEGG analysis

**Figure 3:**
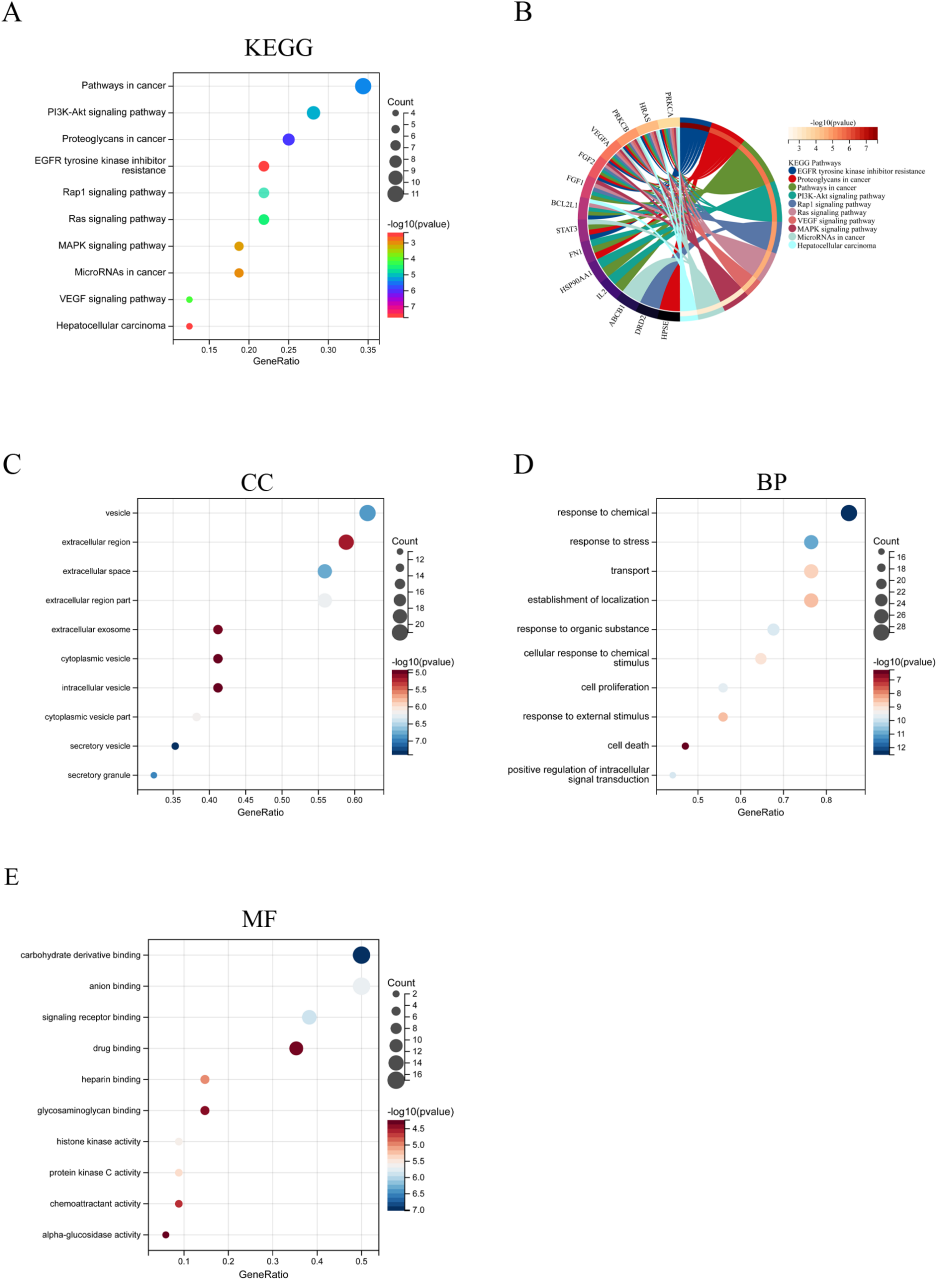
GO and KEGG analysis of Common genes between STA and HCC. **(A)** KEGG analysis results. **(B)** KEGG results circular diagram. **(C)** CC analysis results. **(D)** BP analysis results. **(E)** MF analysis results.

GO and KEGG analyses were conducted on the 34 targets of STA for liver cancer treatment. The KEGG analysis revealed several significant pathways, including pathways in cancer, the PI3K-Akt signaling pathway, proteoglycans in cancer, EGFR tyrosine kinase inhibitor resistance, the Rap1 signaling pathway, the Ras signaling pathway, the MAPK signaling pathway, microRNAs in cancer, the VEGF signaling pathway, and hepatocellular carcinoma (**Figures 3A, B**). In terms of cellular components (CC), the results highlighted several regions such as vesicles, the extracellular region, extracellular space, parts of the extracellular region, extracellular exosomes, cytoplasmic vesicles, intracellular vesicles, parts of cytoplasmic vesicles, secretory vesicles, and secretory granules (**Figure 3C**). For biological processes (BP), the findings included responses to chemicals, stress, transport, localization establishment, responses to organic substances, cellular responses to chemical stimuli, cell proliferation, responses to external stimuli, cell death, and positive regulation of intracellular signal transduction (**Figure 3D**). Regarding molecular functions (MF), the results indicated several activities, including carbohydrate derivative binding, anion binding, signaling receptor binding, drug binding, heparin binding, glycosaminoglycan binding, histone kinase activity, protein kinase C activity, chemoattractant activity, and alpha-glucosidase activity (**Figure 3E**).

### Expression of hub genes in liver cancer and their associations with angiogenesis, tumor inflammation, tumor proliferation, and EMT

**Figure 4:**
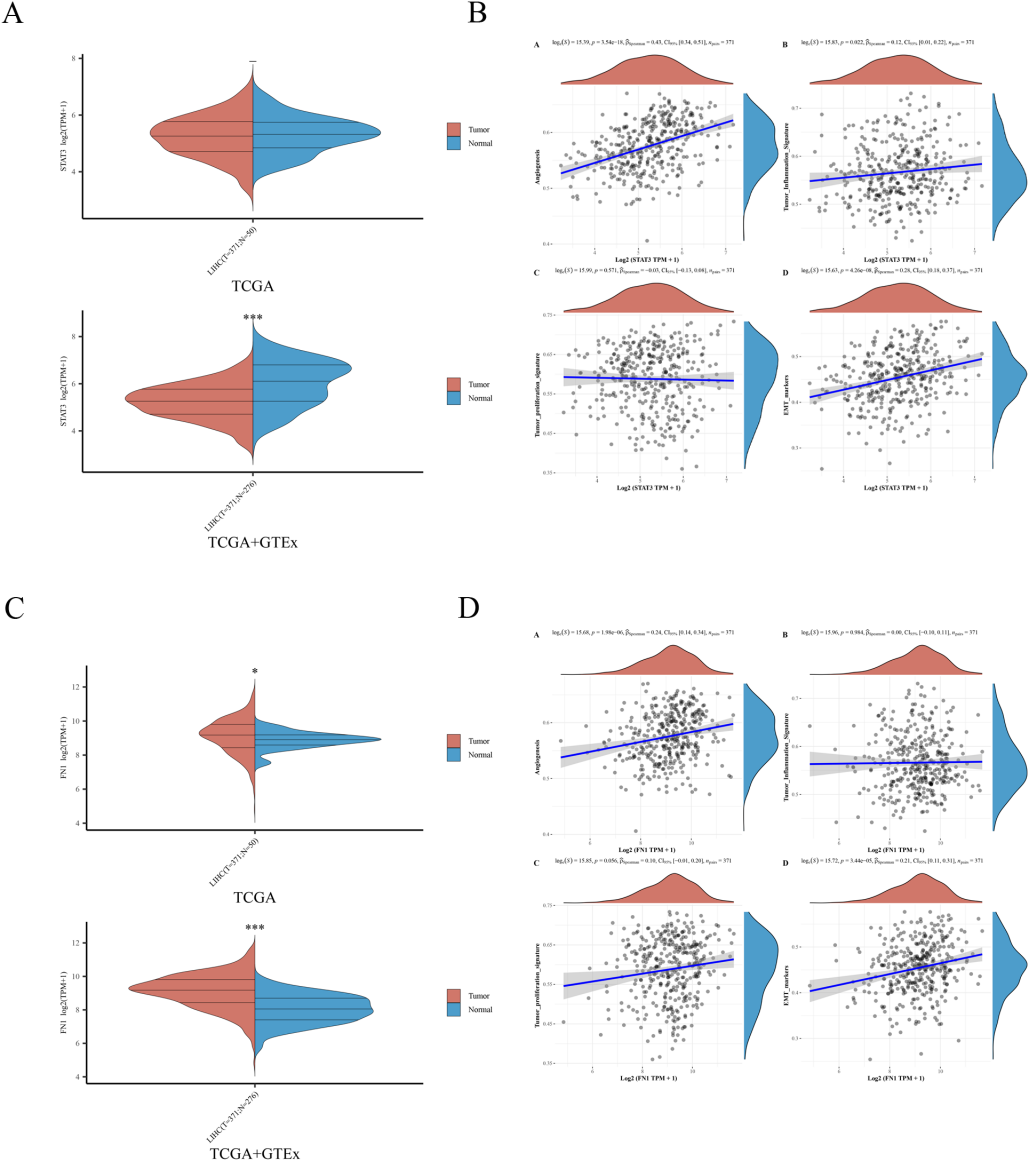
Expression of 2 hub genes in HCC and their association with angiogenesis, tumor inflammation, tumor proliferation, and EMT. **(A)** Expression of STAT3 in HCC samples. **(B)** Relationship between STAT3 and angiogenesis, tumor inflammation, tumor proliferation, EMT. **(C)** Expression of FN1 in HCC samples. **(D)** Relationship between FN1 and angiogenesis, tumor inflammation, tumor proliferation, EMT.

After identifying STAT3 and FN1 as hub genes for STA treatment of liver cancer, we performed single-gene analyses on these genes. STAT3 was found to be downregulated in liver cancer patients, which contradicts the majority of current research (**Figure 4A**) (14, 15). This discrepancy may stem from variations in time points or disease stages among the studies, resulting in dynamic changes in STAT3 expression. Notably, STAT3 showed positive correlations with angiogenesis (p=3.54e-18), tumor inflammation (p=0.022), and epithelial-mesenchymal transition (EMT) (p=4.26e-08), but no correlation with tumor proliferation (p=0.571) (**Figure 4B**). In contrast, FN1 was upregulated in liver cancer patients (**Figure 4C**) and positively correlated with angiogenesis (p=1.98e-06) and EMT (p=3.44e-05), but not associated with tumor inflammation (p=0.984) or tumor proliferation (p=0.056) (**Figure 4D**). These findings suggest that STAT3 and FN1 are potential targets for liver cancer treatment, supporting their role as hub genes in STA treatment.

### Prognostic analysis of hub genes in liver cancer

**Figure 5:**
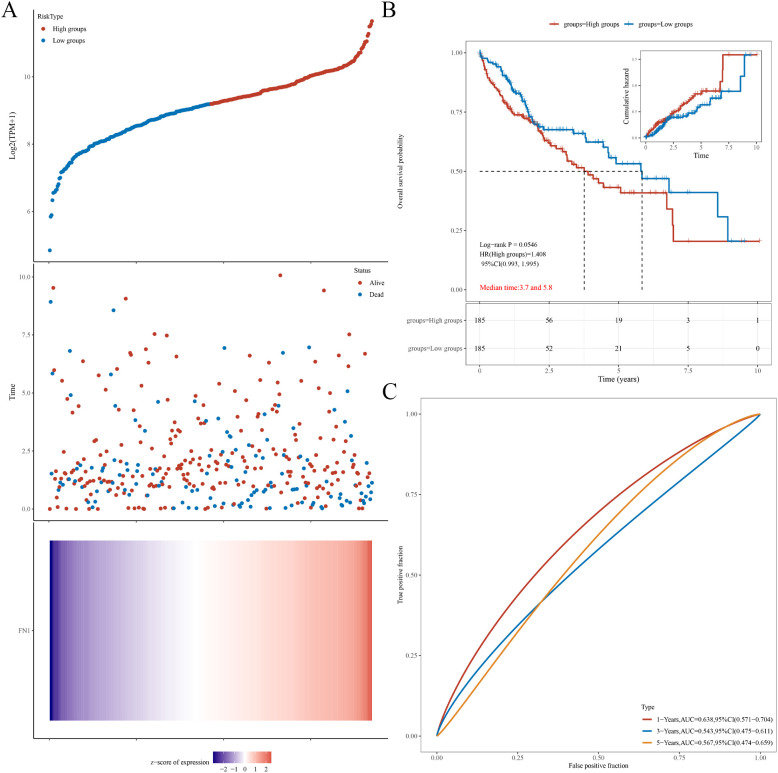
Prognostic analysis of FN1 in HCC. **(A)** Relationship between FN1 expression and survival time and status in TCGA data. **(B)** KM survival curve of FN1 in TCGA data. **(C)** ROC curves and AUC values of FN1 at different time points.

The hazard ratio (HR) quantifies the relative risk between the high expression group and the low expression group. An HR greater than 1 signifies that the gene serves as a risk factor, with higher expression levels associated with a poorer prognosis. Conversely, an HR less than 1 implies that the gene functions as a protective factor, where higher expression levels are linked to a more favorable prognosis. The area under the curve (AUC) metric, used for model evaluation, ranges from 0 to 1, with higher values denoting superior predictive performance of the model. An AUC of 0.5 suggests the model is nearly random and lacks meaningful predictive power; typically, a prognostic model should achieve an AUC above 0.7. The prognostic analysis for STAT3 showed an HR of 0.916, with no statistically significant difference in overall survival probability between high and low expression groups (p=0.62) (**Supplementary Figures S1A, B**). The AUC values for 1, 3, and 5 years were close to 0.5, indicating that the model is ineffective (**Supplementary Figure S1C**). For FN1, the analysis revealed an HR of 1.408, also showing no statistically significant difference in overall survival probability between expression groups (p=0.0546) (**Figures 5A, B**), with AUC values similarly close to 0.5, indicating a lack of meaningful predictive power (**Figure 5C**). In conclusion, STAT3 and FN1 do not provide significant prognostic value, while the FN1 low-expression group had a better overall survival probability.

### Analysis of the correlation between FN1 and STAT3 and differential gene analysis of FN1 high and low expression groups

**Figure 6:**
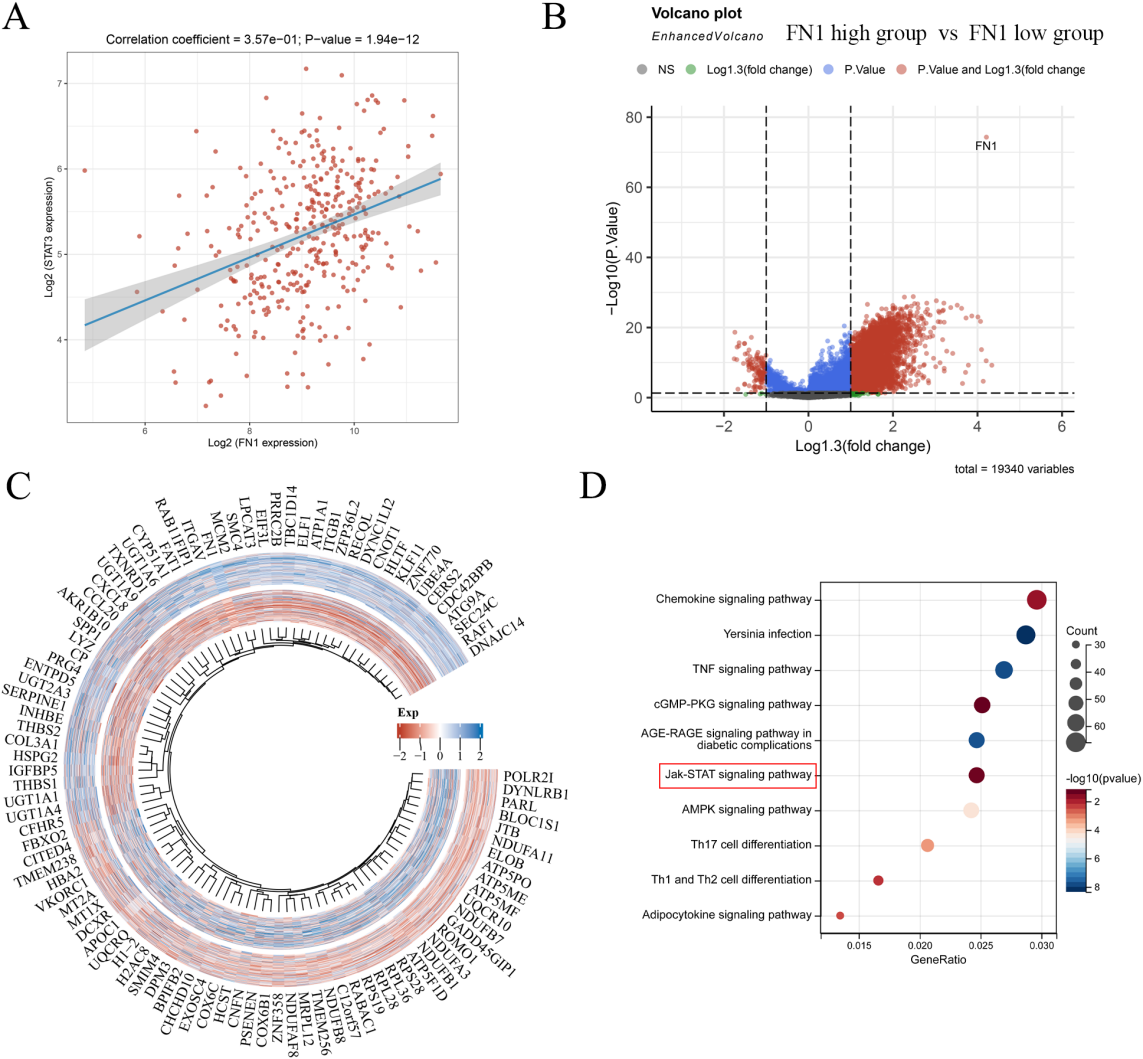
Correlation between FN1 and STAT3 and differential gene analysis of FN1 high and low expression groups. **(A)** Correlation analysis of FN1 and STAT3 in liver cancer. **(B)** Volcano plot of FN1 high and low expression groups. **(C)** Heatmap of differential genes in FN1 high and low expression groups. **(D)** KEGG functional enrichment of differential genes in FN1 high and low expression groups.

In TCGA liver cancer data, we found a positive correlation between the expression of FN1 and STAT3 (**Figure 6A**). Subsequently, we divided liver cancer patients into FN1 high expression group and FN1 low expression group, and there were no statistically significant differences in age, race, gender, and pTNM stage between the two groups (**Supplementary Figure S2**). Differential gene analysis of these two groups revealed 5018 upregulated genes and 123 downregulated genes (**Figures 6B, C**). After performing KEGG functional enrichment analysis on these differential genes, they were found to be enriched in the JAK-STAT signaling pathway (**Figure 6D**). Therefore, there is a certain association between FN1 and STAT3 in liver cancer.

### Molecular docking verification of STA binding to hub genes

**Figure 7:**
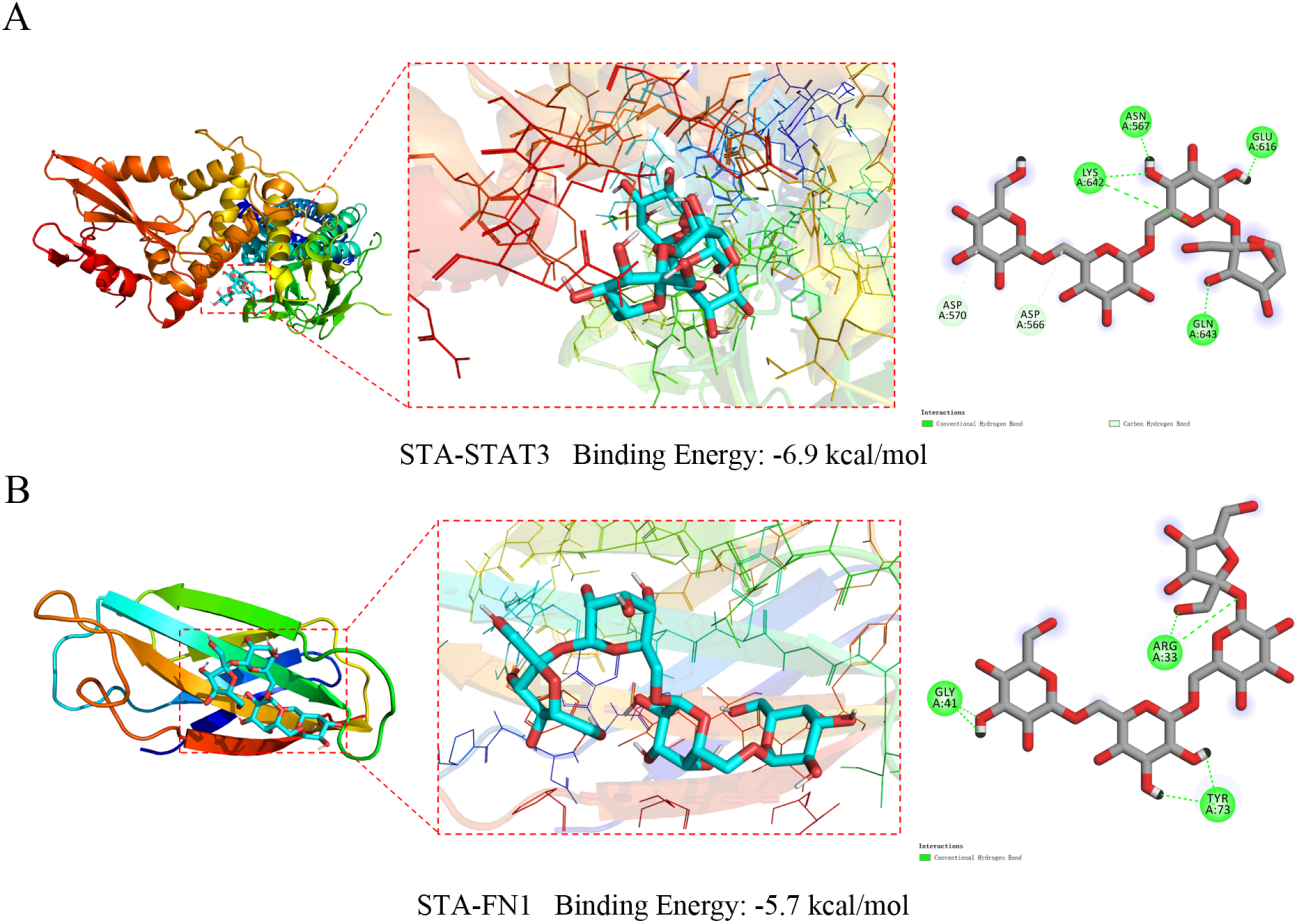
Molecular docking results of STA with hub genes. **(A)** Molecular docking results of STA with STAT3. **(B)** Molecular docking results of STA with FN1.

When the binding energy is <0 kJ/mol, it indicates that the ligand and receptor can bind spontaneously. Lower values signify stronger binding and greater affinity. The binding energy of STA to STAT3 was -6.9 kcal/mol, with conventional hydrogen bonds formed with ASN567, GLU616, LYS642, and GLN643, as well as carbon hydrogen bonds with ASP570 and ASP566 (**Figure 7A**). For STA-FN1, the binding energy was -5.7 kcal/mol, involving conventional hydrogen bonds with ARG33, GLY41, and TYR73 (**Figure 7B**). These results indicate stable binding between STA and the hub genes STAT3 and FN1.

### *In vitro* experimental verification of STA regulation on STAT3 and FN1

**Figure 8:**
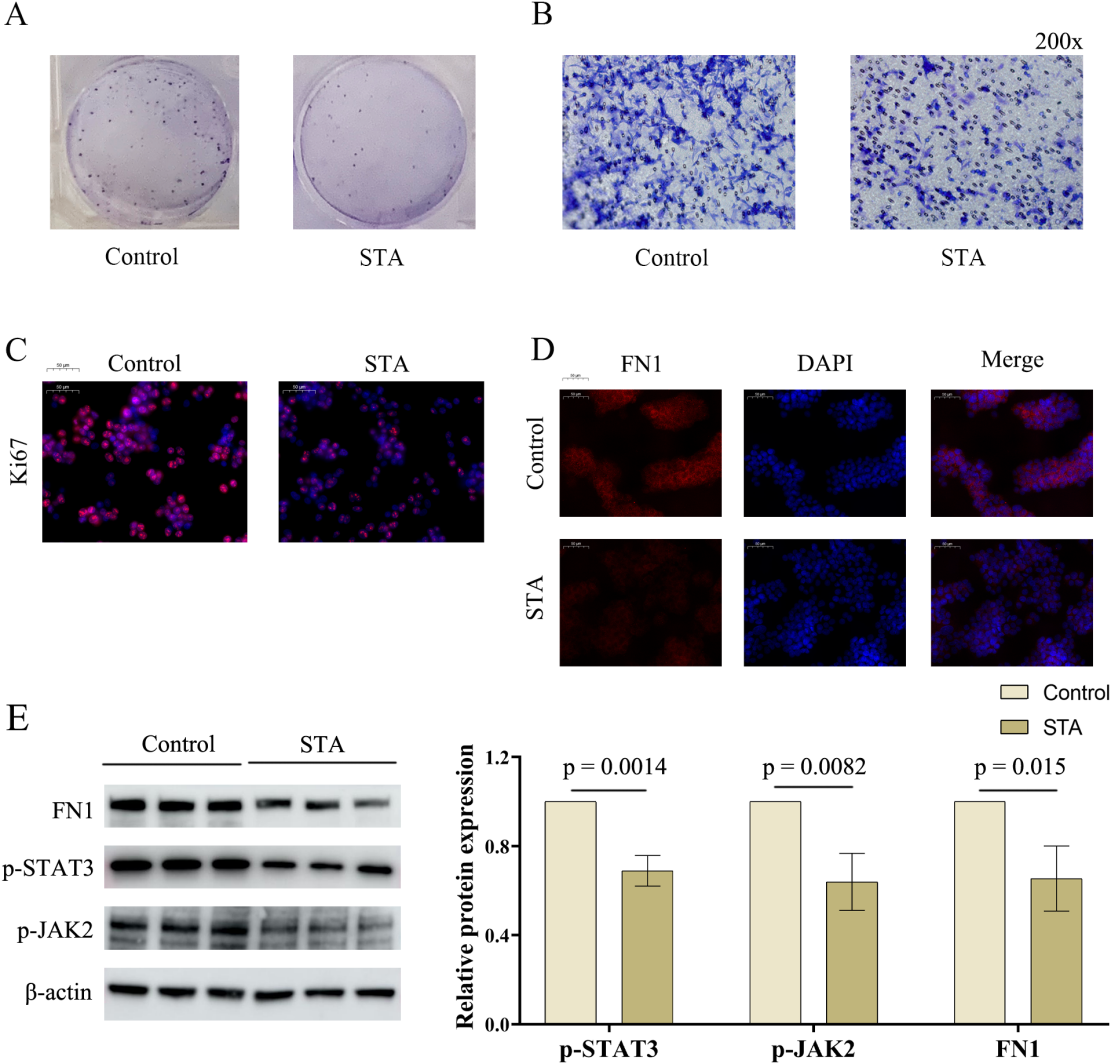
Effects of STA on HCC cells and regulation of hub genes. **(A)** Effect of STA on HCC cell proliferation. **(B)** Effect of STA on HCC cell migration. **(C)** Immunofluorescence experiment of Ki67. **(D)** Immunofluorescence experiment of FN1. **(E)** Effect of STA on protein expression of FN1, p-STAT3, and p-JAK2 in HCC cells.

After network pharmacology and single-gene analysis, we preliminarily identified STAT3 and FN1 as the molecular targets through which STA exerts its effect on liver cancer. In the colony formation assay, STA significantly inhibited cancer cell proliferation (**Figure 8A**). Similarly, the Transwell migration assay showed that STA reduced hepatocyte migration (**Figure 8B**). In the immunofluorescence experiment, the expression of Ki67 (a tumor proliferation marker) was inhibited by STA (**Figure 8C**). These findings confirm the anti-liver cancer effect of STA. In the immunofluorescence assay for FN1, STA suppressed its expression (**Figure 8D**). In addition, Western blot experiments also demonstrated that STA can effectively downregulate the expression of p-STAT3, p-JAK2, and FN1 (**Figure 8E**). In conclusion, STA may exert its therapeutic effect on cancer by regulating JAK-STAT signaling pathway and FN1.

## Discussion

This study systematically investigated the potential molecular mechanisms of STA in the treatment of HCC, identifying STAT3 and FN1 as key targets. Through multi-level bioinformatics analysis and experimental validation, we elucidated the mechanisms underlying STA’s action in HCC treatment, providing both theoretical support and experimental evidence. Initially, we utilized the Swiss Target Prediction database to screen for potential STA targets, subsequently identifying HCC-related targets by integrating data from GeneCards, OMIM, the Therapeutic Target Database, and PHARMGKB. Venn diagram analysis revealed 34 common targets between STA and HCC. Further construction of the PPI network using the STRING database, along with topological analysis via Cytoscape software, highlighted STAT3 and FN1 as hub genes. These genes are crucial in the occurrence, progression, and treatment of HCC.

STAT3, a signal transducer and transcription activator, exhibits significant activation across a range of cancers and plays a pivotal role in promoting cell proliferation, inhibiting apoptosis, facilitating angiogenesis, and enabling immune evasion (16–18). Research has shown that the overexpression of zinc finger protein 263 (ZNF263) markedly promotes the proliferation, invasion, migration, and EMT of colorectal cancer (CRC) cells, concomitantly elevating STAT3 expression and mRNA stability. Importantly, the knockdown or overexpression of STAT3 can significantly counteract the effects of ZNF263 on CRC cells (19). Additionally, FN1 is pivotal in the tumor microenvironment, mediating cell adhesion, migration, and signal transduction (20–22). It augments the migratory and invasive capacities of tumor cells through the modulation of the EMT process. Additionally, FN1 contributes to tumor microenvironment remodeling through interactions with other extracellular matrix proteins, further supporting tumor growth and dissemination (23). Our research suggests that these genes may play crucial roles in STA’s treatment of HCC by influencing tumor cell proliferation and migration, thereby exerting anti-tumor effects.

GO and KEGG pathway analyses provided additional insights into the biological functions and signaling pathways associated with the 34 targets in HCC. The results indicated that these targets are primarily enriched in pathways related to cell proliferation, apoptosis, angiogenesis, and immune response, which align closely with the pathophysiological processes of HCC (24, 25). Notably, the JAK/STAT, PI3K/AKT, and MAPK signaling pathways are well-recognized for their critical roles in HCC development (26–28). STA may inhibit tumor cell growth and dissemination by modulating these signaling pathways.

To assess the clinical relevance of STAT3 and FN1 in HCC, we performed single-gene expression and prognostic analyses. We examined the relationships between these genes and various tumor biological processes, discovering that they are closely linked to angiogenesis, tumor inflammation, tumor proliferation, and EMT. Molecular docking experiments demonstrated that STA exhibits a high binding affinity for STAT3 and FN1, further supporting the notion of these genes as potential targets for STA. Although STAT3 and FN1 play different roles in liver cancer, there may be some connection between them. The activation of STAT3 can influence the remodeling of the extracellular matrix and intercellular interactions through various signaling pathways, while FN1, as an important component of the extracellular matrix, may be affected in these processes (29). Furthermore, the overactivation of STAT3 is often associated with inflammatory responses in the tumor microenvironment, and FN1 may also participate in these inflammatory responses during extracellular matrix remodeling (30). By analyzing the common signaling pathways of STAT3 and FN1, their potential connection in liver cancer can be further revealed. For example, the PI3K-Akt and JAK-STAT signaling pathways may be key pathways in which STAT3 and FN1 are jointly involved, playing significant roles in the occurrence and development of liver cancer (31, 32). Through in-depth studies of these pathways, a better understanding of the synergistic mechanisms of STAT3 and FN1 in liver cancer can be achieved, providing new targets and strategies for the treatment of liver cancer (33, 34). *In vitro* experiments confirmed the regulatory effects of STA on p-STAT3, p-JAK and FN1, indicating that STA can effectively inhibit the expression and function of these genes, thereby exerting anti-tumor effects.

In conclusion, this study offers a comprehensive exploration of the molecular mechanisms through which STA may treat HCC, identifying STAT3 and FN1 as key targets and validating their clinical relevance and potential for application. These findings not only establish a theoretical foundation for STA as a targeted therapeutic agent for HCC but also open new avenues for targeted therapy research in this area. Future investigations should focus on the safety and efficacy of STA in clinical settings and develop combination treatment strategies centered on these targets, with the ultimate goal of improving survival rates and quality of life for HCC patients.

## Conclusion

This study, utilizing bioinformatics and experimental validation, has demonstrated that STA may exert its anti-hepatocellular carcinoma effects through multiple targets and pathways, particularly by regulating STAT3 and FN1. These findings provide compelling scientific evidence for the potential application of STA as a treatment for liver cancer. However, further *in vivo* experiments and clinical studies are needed to confirm the specific therapeutic efficacy and safety of STA in the management of liver cancer.

## Data Availability

The original contributions presented in the study are included in the article/Supplementary Material. Further inquiries can be directed to the corresponding authors.
