# Supplementary material for: Identification of key molecular targets for stachyose in hepatocellular carcinoma: focus on STAT3 and FN1
Source: Front Oncol. 2025 Jul 23;15:1576449. doi: 10.3389/fonc.2025.1576449 (PMC12325021; doi:10.3389/fonc.2025.1576449)

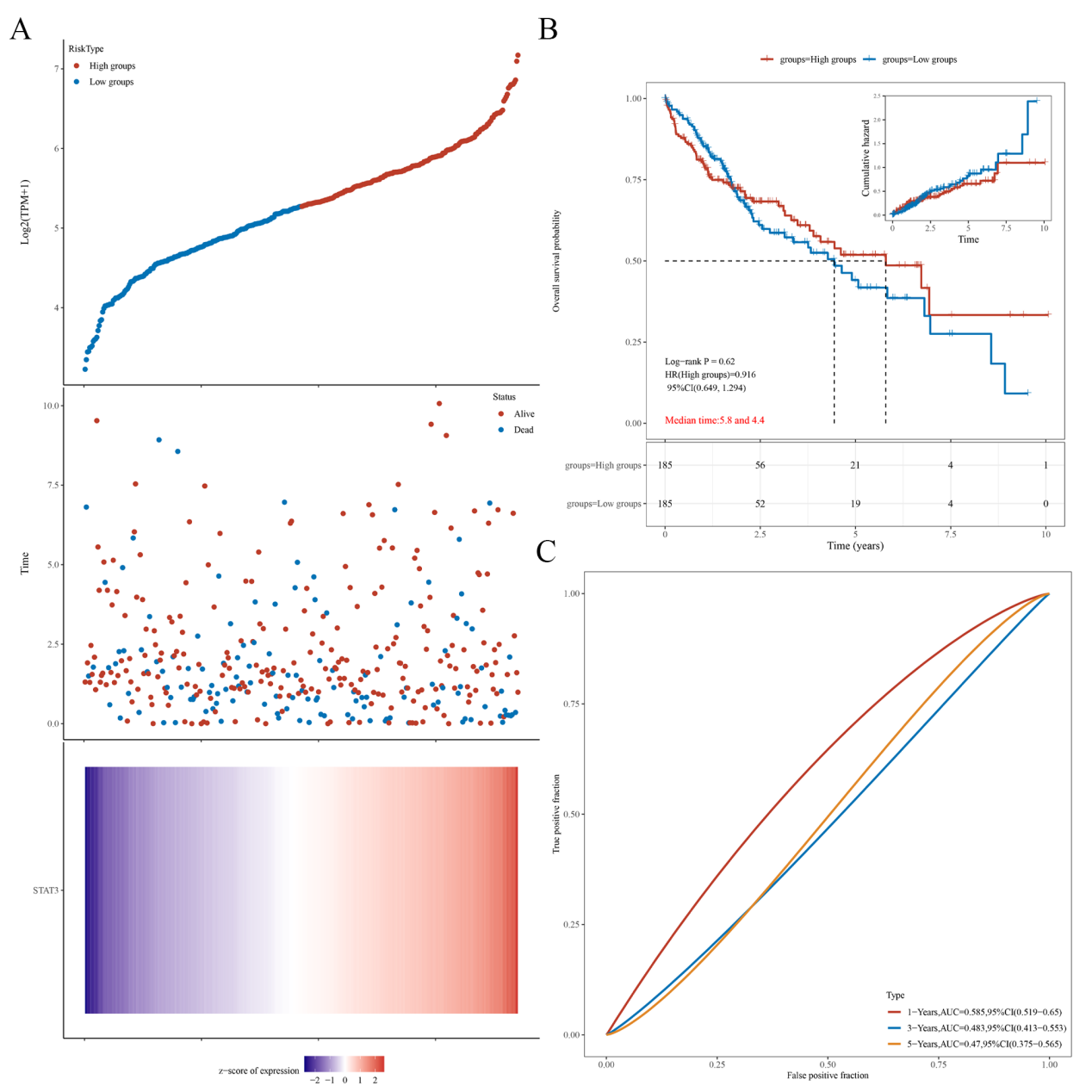


Figure S1. Prognostic analysis of STAT3 in hepatocellular carcinoma.

(A) Relationship between STAT3 expression and survival time and status in TCGA data. (B) Kaplan-Meier survival curves for STAT3 expression in TCGA data. (C) Time-dependent ROC curves and AUC values for STAT3 expression.


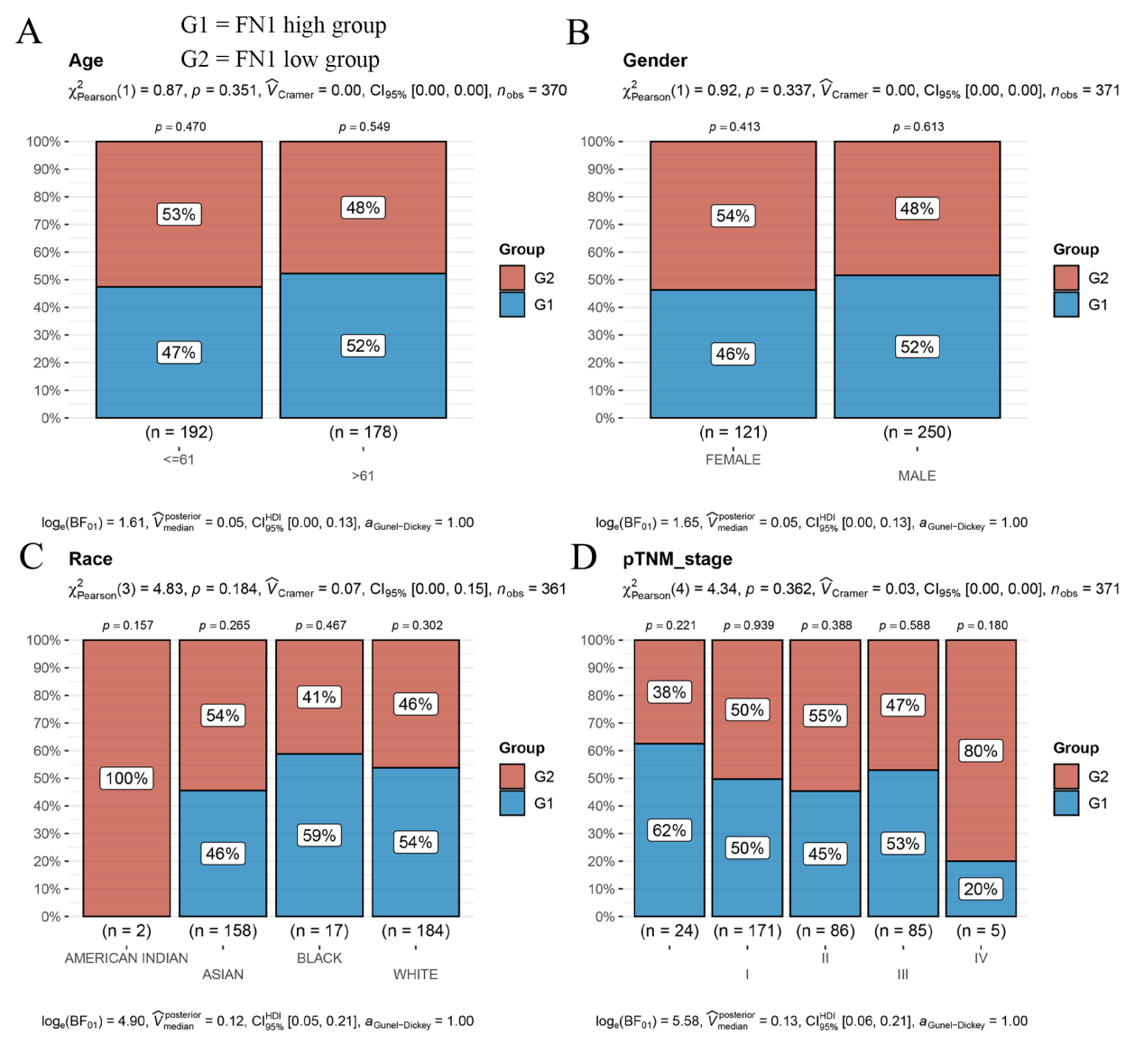


Figure S2. Clinical information of FN1 high and low expression groups in TCGA liver cancer patients.

1. Age statistics of FN1 high and low expression groups. (B) Gender statistics of FN1 high and low expression groups. (C) Racial statistics of FN1 high and low expression groups. (D) pTNM stage statistics of FN1 high and low expression groups.

Table S1. Target Prediction of Hepatocellular Carcinoma and STA in Various Databases.

| GeneCards （Relevance score>=10） | OMIM | TTD | PHARMGKB | Liver Neoplasms | Stachyose |
| --- | --- | --- | --- | --- | --- |
| MSH6 | ALPL | EGFR | HULC | MSH6 | CDK1 |
| MSH2 | RNF220 | FGFR1 | CT47A12 | MSH2 | VEGFA |
| MLH1 | PKLR | FGFR2 | CT45A8 | MLH1 | FGF1 |
| PMS2 | FMO1 | HDAC1 | CT45A9 | PMS2 | FGF2 |
| PKHD1 | FMO4 | JAK-2 | CT45A10 | PKHD1 | HPSE |
| TP53 | NBAS | mTOR | CT45A7 | TP53 | LGALS4 |
| CTNNB1 | APOB | KIT | CT47A1 | CTNNB1 | LGALS8 |
| PKD1 | HADHA | KDR | CT47A11 | PKD1 | PSEN2 |
| KRAS | FABP1 | IGF1R | CT47A4 | KRAS | HTR2B |
| PTEN | AGXT | PIK3CG | CT47A6 | PTEN | ADRA2A |
| CDKN2A | DNAJB11 | PDGFRB | CT47B1 | CDKN2A | ADRA2C |
| APC | HSD17B13 | MET | CT47A10 | APC | ADRA2B |
| PIK3CA | LEAP2 | S1PR1 | CT47A3 | PIK3CA | DRD1 |
| EGFR | LARS1 | CTLA-4 | CT47A5 | EGFR | DRD2 |
| BRAF | DDX41 | STAT3 | CT47A7 | BRAF | ADRA1D |
| MET | HLA-B | TERT | CT47A8 | MET | HTR2A |
| AKT1 | COX7A2 | CSF2 | CT47A9 | AKT1 | HTR2C |
| TERT | SEC63 | HB | CT47A3 | TERT | DRD3 |
| H19 | ARG1 | IDH1 | CT45A2 | H19 | CYP2D6 |
| ERBB2 | RINT1 | PD-L1 | CT45A6 | ERBB2 | HTR6 |
| KIT | CDH17 | PD-1 | CT45A5 | KIT | ADRA1A |
| BRCA2 | FOCAD | TLR7 | UCA1 | BRCA2 | HTR1B |
| CCND1 | LRP5 | TGFB1 | COLCA2 | CCND1 | HSP90AA1 |
| ATM | CPT1A | VEGFA | PRAC1 | ATM | LGALS3 |
| MEG3 | ALG8 | TOP2 | CAGE1 | MEG3 | RORC |
| MIR21 | NAFLD2 | hDNA | CT45B1P | MIR21 | TRPV1 |
| SMAD4 | GYS2 | FGFR3 | CT83 | SMAD4 | AMY2A |
| EPCAM | GLS2 | TERT | KIAA0100 | EPCAM | STAT3 |
| HRAS | MARS1 | ADORA3 | LTO1 | HRAS | AMY1A |
| SEC63 | RNASE2 | AURKA | PAGE5 | SEC63 | SLC6A2 |
| NRAS | PYGL | CSNK2A1 | CTAG2 | NRAS | GLRA1 |
| NF1 | PHKG2 | TGFBR1 | XAGE3 | NF1 | GLRA2 |
| BRCA1 | PHKB | FLT-4 | NTPCR | BRCA1 | SSTR5 |
| TSC2 | PRKCSH | TP53 | SDCCAG8 | TSC2 | SSTR2 |
| PKD2 | PFKL | FGFR4 | LETMD1 | PKD2 | SSTR4 |
| MIR7-3HG | NAFLD1 | TRAIL-R2 | GREB1 | MIR7-3HG | SSTR1 |
| RET | TRMU | BSG | SMIM22 | RET | SSTR3 |
| STAT3 | SLC25A6 | DKK1 | DERPC | STAT3 | OPRK1 |
| PDGFRA | PHKA2 | FGFR4 | DSCR8 | PDGFRA | ADORA1 |
| CDH1 |  | KIF11 | CTAGE1 | CDH1 | IL2 |
| LRP5 |  | MKNK2 | DDX53 | LRP5 | CA7 |
| PRKCSH |  | MKNK1 | CT62 | PRKCSH | CA6 |
| MALAT1 |  | SPHK2 | VENTXP1 | MALAT1 | CA4 |
| TSC1 |  | AFP | PCA3 | TSC1 | CA13 |
| IL6 |  | EGFR vIII | BCAR3 | IL6 | CA5B |
| FGFR1 |  | MSLN | ANKRD45 | FGFR1 | CA5A |
| STK11 |  | MUC1 | GAGE4 | STK11 | PPM1A |
| GNAS |  | MYCBP | GAGE5 | GNAS | MAG |
| JAK2 |  | ENG | GAGE6 | JAK2 | PTAFR |
| MYC |  | FACT | C20orf85 | MYC | AKR1B1 |
| TRMU |  | GPC3 | C1orf74 | TRMU | ADORA2A |
| NOTCH1 |  | RORG | C4orf46 | NOTCH1 | FPGS |
| CASP8 |  | EPCAM | CASC2 | CASP8 | SELP |
| MARS1 |  | PROM1 | CTAG1B | MARS1 | P2RY4 |
| ALK |  | CRBN | GAGE7 | ALK | HRAS |
| HNF4A |  | MIR34 | HEPN1 | HNF4A | P2RY2 |
| HNF1A |  | CD80/PD-L1 PPI | SNCG | HNF1A | P2RY6 |
| IDH1 |  | PD-1/PD-L1 PPI | ARMC3 | IDH1 | RNASE2 |
| TNF |  | RORA | HEATR6 | TNF | RNASE1 |
| MIR17 |  | KMT5A | TFDP1 | MIR17 | MLNR |
| MIR34A |  | DDEFL1 | BAGE | MIR34A | FOLH1 |
| HULC |  | IL27 | CTAG1A | HULC | EPHX2 |
| PNPLA3 |  | MIR191 | ANKRD30BP2 | PNPLA3 | ASNS |
| FGFR2 |  | IFNA | SPANXN3 | FGFR2 | GBA |
| FGFR3 |  | RAR | SPANXN2 | FGFR3 | RNASEL |
| SRC |  | CEBPA? | SPANXN5 | SRC | FN1 |
| HNF1B |  | FGFR | BAGE5 | HNF1B | VDR |
| HOTAIR |  | HLA-A02/AFP | PBOV1 | HOTAIR | CA2 |
| PPARG |  | PK | SPANXB1 | PPARG | CA1 |
| RB1 |  | S1PR | FAM168A | RB1 | CA9 |
| CHEK2 |  | CSK | XAGE5 | CHEK2 | SI |
| VHL |  | CSNK2 | CHD1L | VHL | LGALS9 |
| PTPN11 |  | STK | MAGEB3 | PTPN11 | PTPN1 |
| GAS5 |  | TCR | RMC1 | GAS5 | IGF2R |
| GYS2 |  | Wnt pathway | DPPA2 | GYS2 | BCL2L1 |
| KRT7 |  |  | XAGE2 | KRT7 | CA12 |
| ALB |  |  | CDK2AP2 | ALB | HSD11B2 |
| MIR122 |  |  | SPAG9 | MIR122 | HSD11B1 |
| TGFB1 |  |  | KNL1 | TGFB1 | ATP1A1 |
| GANAB |  |  | GKN2 | GANAB | KCNH2 |
| ESR1 |  |  | TCIM | ESR1 | OPRM1 |
| IDH2 |  |  | LUZP4 | IDH2 | ABCB1 |
| INS |  |  | CT55 | INS | ABCB11 |
| FH |  |  | RBM46 | FH | CA14 |
| MIR125A |  |  | SPANXN4 | MIR125A | PPM1B |
| NBN |  |  | SPANXN1 | NBN | PPP1CC |
| BCL2 |  |  | LYPD6B | BCL2 | PPP2CA |
| BAP1 |  |  | MAGEA9 | BAP1 | PPP2R5A |
| ARID1A |  |  | POTEC | ARID1A | TYR |
| PVT1 |  |  | BRCA2 | PVT1 | GAA |
| MIR126 |  |  | NXF2 | MIR126 | FDFT1 |
| HFE |  |  | SAGE1 | HFE | OGA |
| MUC1 |  |  | BAGE4 | MUC1 | PRKCG |
| SERPINA1 |  |  | GAGE8 | SERPINA1 | PRKCD |
| WT1 |  |  | BAGE2 | WT1 | PRKCA |
| MIR155 |  |  | ACTL8 | MIR155 | PRKCB |
| MIR221 |  |  | BLID | MIR221 | PRKCE |
| MIR31 |  |  | MAGEB4 | MIR31 | PRKCH |
| MIR30A |  |  | SPANXD | MIR30A | PRKCQ |
| CREBBP |  |  | CCDC33 | CREBBP | SLC5A2 |
| CDKN1B |  |  | POTEA | CDKN1B |  |
| MIR27A |  |  | GAGE1 | MIR27A |  |
| NFE2L2 |  |  | CSAG1 | NFE2L2 |  |
| MIR223 |  |  | CCDC110 | MIR223 |  |
| MEN1 |  |  | HID1 | MEN1 |  |
| MAP2K1 |  |  | BAGE3 | MAP2K1 |  |
| XIST |  |  | CCDC62 | XIST |  |
| MIR200A |  |  | ROPN1 | MIR200A |  |
| RAF1 |  |  | POTEG | RAF1 |  |
| NBAS |  |  | GAGE2A | NBAS |  |
| MIR22 |  |  | TULP2 | MIR22 |  |
| MDM2 |  |  | HSPB9 | MDM2 |  |
| CDK4 |  |  | PRAC2 | CDK4 |  |
| MTOR |  |  | CENPW | MTOR |  |
| SMO |  |  | FMR1NB | SMO |  |
| MIR200B |  |  | MAGEC2 | MIR200B |  |
| FAS |  |  | ICE2 | FAS |  |
| TUG1 |  |  | TENT5D | TUG1 |  |
| MIR143 |  |  | POTED | MIR143 |  |
| AR |  |  | POTEH | AR |  |
| MIR146A |  |  | VOPPI | MIR146A |  |
| TGFBR2 |  |  | MAGEA3 | TGFBR2 |  |
| AXIN1 |  |  | FATE1 | AXIN1 |  |
| GPT |  |  | MAGEC3 | GPT |  |
| MIR145 |  |  | CSAG2 | MIR145 |  |
| PTCH1 |  |  | GOLGA6L2 | PTCH1 |  |
| KRT20 |  |  | HORMAD1 | KRT20 |  |
| NTRK1 |  |  | RTL6 | NTRK1 |  |
| AFP |  |  | GPATCH2 | AFP |  |
| RAD50 |  |  | MAGEA10 | RAD50 |  |
| DDX41 |  |  | CIP2A | DDX41 |  |
| NF2 |  |  | SPANXC | NF2 |  |
| MIR214 |  |  | SPANXA1 | MIR214 |  |
| MIR203A |  |  | MAGEA2 | MIR203A |  |
| CDKN2B-AS1 |  |  | POTEE | CDKN2B-AS1 |  |
| SDHB |  |  | PRSS54 | SDHB |  |
| UCA1 |  |  | OCIAD2 | UCA1 |  |
| SDHD |  |  | ODF3 | SDHD |  |
| IFNG |  |  | LIPI | IFNG |  |
| ICAM1 |  |  | CPXCR1 | ICAM1 |  |
| GPC3 |  |  | SLC49A4 | GPC3 |  |
| MIRLET7C |  |  | ENTR1 | MIRLET7C |  |
| PALB2 |  |  | PASD1 | PALB2 |  |
| RXRA |  |  | SPINK7 | RXRA |  |
| MIR222 |  |  | BRMS1L | MIR222 |  |
| ERBB4 |  |  | LRATD2 | ERBB4 |  |
| PDGFRB |  |  | OVCA2 | PDGFRB |  |
| HIF1A |  |  | AGR3 | HIF1A |  |
| MIR15A |  |  | ODF1 | MIR15A |  |
| MIR20A |  |  | MAGEB6 | MIR20A |  |
| CYP3A4 |  |  | DCAF12 | CYP3A4 |  |
| DICER1 |  |  | DEPDC1B | DICER1 |  |
| ALG8 |  |  | SLCO6A1 | ALG8 |  |
| YAP1 |  |  | TEX101 | YAP1 |  |
| IL10 |  |  | TBC1D3 | IL10 |  |
| MIR141 |  |  | PLAC1 | MIR141 |  |
| SMARCB1 |  |  | GREB1L | SMARCB1 |  |
| LINC-ROR |  |  | LDOC1 | LINC-ROR |  |
| MIR29C |  |  | MAGEC1 | MIR29C |  |
| IL1B |  |  | TMEM108 | IL1B |  |
| IGF2 |  |  | ZNF165 | IGF2 |  |
| NR1H4 |  |  | SPATA19 | NR1H4 |  |
| LOC126806658 |  |  | SSX2 | LOC126806658 |  |
| MIR148A |  |  | SPA17 | MIR148A |  |
| LOC126859690 |  |  | TMEFF1 | LOC126859690 |  |
| NEAT1 |  |  | C14orf93 | NEAT1 |  |
| MIR18A |  |  | XAGE1A | MIR18A |  |
| MIR183 |  |  | NLRP4 | MIR183 |  |
| KCNQ1OT1 |  |  | ANKRD30A | KCNQ1OT1 |  |
| MUTYH |  |  | PNMA3 | MUTYH |  |
| HGF |  |  | MAGEB2 | HGF |  |
| MIR195 |  |  | ACRBP | MIR195 |  |
| SMARCA4 |  |  | RHOXF2 | SMARCA4 |  |
| MIR132 |  |  | MAGEA1 | MIR132 |  |
| FOXM1 |  |  | EPPIN | FOXM1 |  |
| PYGL |  |  | SLC4A1AP | PYGL |  |
| CCAT1 |  |  | COX6B2 | CCAT1 |  |
| CDKN1A |  |  | SPEF2 | CDKN1A |  |
| MIR150 |  |  | ECRG4 | MIR150 |  |
| IGF2R |  |  | PRSS50 | IGF2R |  |
| MIR142 |  |  | ODF4 | MIR142 |  |
| VEGFA |  |  | MORC1 | VEGFA |  |
| PHKA2 |  |  | BRINP1 | PHKA2 |  |
| MIR29A |  |  | TMEFF2 | MIR29A |  |
| MIR151A |  |  | CALR3 | MIR151A |  |
| MIR139 |  |  | FAM133A | MIR139 |  |
| UGT1A1 |  |  | POTEB | UGT1A1 |  |
| ABCB4 |  |  | MAGEB5 | ABCB4 |  |
| F2 |  |  | HELZ | F2 |  |
| NTRK2 |  |  | MAGED2 | NTRK2 |  |
| MIR205 |  |  | THEG | MIR205 |  |
| MIR140 |  |  | DDX43 | MIR140 |  |
| KRT19 |  |  | MAGEA12 | KRT19 |  |
| BAX |  |  | CEP55 | BAX |  |
| MIRLET7B |  |  | SSX1 | MIRLET7B |  |
| FASLG |  |  | SEMG1 | FASLG |  |
| MIR10B |  |  | MACC1 | MIR10B |  |
| MIR106B |  |  | BRMS1 | MIR106B |  |
| KDR |  |  | MAGEA11 | KDR |  |
| MIR130A |  |  | MAGEA6 | MIR130A |  |
| ABCB1 |  |  | TFDP3 | ABCB1 |  |
| CDKN2B |  |  | DLEC1 | CDKN2B |  |
| MIR23A |  |  | IGSF11 | MIR23A |  |
| ABCB11 |  |  | BCAR3 | ABCB11 |  |
| MIR127 |  |  | BRCA1 | MIR127 |  |
| MIRLET7D |  |  | FTHL17 | MIRLET7D |  |
| HOTTIP |  |  | CNOT9 | HOTTIP |  |
| SDHC |  |  | MAGEA8 | SDHC |  |
| NTRK3 |  |  | LEMD1 | NTRK3 |  |
| MIR23B |  |  | HIC1 | MIR23B |  |
| EZH2 |  |  | PBK | EZH2 |  |
| MIR182 |  |  | DKKL1 | MIR182 |  |
| MIR30E |  |  | MRPS11 | MIR30E |  |
| SLC17A5 |  |  | IL13RA2 | SLC17A5 |  |
| VDR |  |  | LY6K | VDR |  |
| SF3B1 |  |  | SSX2B | SF3B1 |  |
| MIR93 |  |  | CRISP2 | MIR93 |  |
| CYP2E1 |  |  | NOL4 | CYP2E1 |  |
| PRKAR1A |  |  | ELOVL4 | PRKAR1A |  |
| MIR200C |  |  | MAGEB1 | MIR200C |  |
| MIR25 |  |  | SYCE1 | MIR25 |  |
| IL2 |  |  | FILIP1L | IL2 |  |
| ABCC2 |  |  | TEX14 | ABCC2 |  |
| MIR212 |  |  | OTOA | MIR212 |  |
| EP300 |  |  | LDHC | EP300 |  |
| CALR |  |  | BLCAP | CALR |  |
| MIR26A1 |  |  | TEX15 | MIR26A1 |  |
| ERBB3 |  |  | SPAG4 | ERBB3 |  |
| NOTCH2 |  |  | ADAM29 | NOTCH2 |  |
| ATP7B |  |  | LSM1 | ATP7B |  |
| MIR99A |  |  | TSSK6 | MIR99A |  |
| CBS |  |  | OCIAD1 | CBS |  |
| JAG1 |  |  | CCAR2 | JAG1 |  |
| BRIP1 |  |  | GPAT2 | BRIP1 |  |
| IGF1 |  |  | AKAP3 | IGF1 |  |
| CYTOR |  |  | ATAD2 | CYTOR |  |
| INSR |  |  | OIP5 | INSR |  |
| MIR107 |  |  | TDRD6 | MIR107 |  |
| MIR181A1 |  |  | MYCL | MIR181A1 |  |
| GLUL |  |  | PRM1 | GLUL |  |
| MIR224 |  |  | PRM2 | MIR224 |  |
| CXCR4 |  |  | TSGA10 | CXCR4 |  |
| KRT18 |  |  | MAGEA4 | KRT18 |  |
| MIR191 |  |  | HIC1 | MIR191 |  |
| CEBPA |  |  | SCA1 | CEBPA |  |
| LRRC56 |  |  | ADAM2 | LRRC56 |  |
| SLC11A2 |  |  | SYCP1 | SLC11A2 |  |
| ENG |  |  | MCC | ENG |  |
| DANCR |  |  | NUF2 | DANCR |  |
| CRP |  |  | TPTE | CRP |  |
| CTLA4 |  |  | MLH1 | CTLA4 |  |
| G6PC1 |  |  | SPO11 | G6PC1 |  |
| MAPK1 |  |  | TDRD1 | MAPK1 |  |
| FLCN |  |  | TSPY1 | FLCN |  |
| MIR125B1 |  |  | IGF2BP3 | MIR125B1 |  |
| PIK3R1 |  |  | NDUFC2 | PIK3R1 |  |
| JUN |  |  | PTEN | JUN |  |
| NFKB1 |  |  | TTK | NFKB1 |  |
| CASC2 |  |  | BRDT | CASC2 |  |
| CALCA |  |  | KIF20B | CALCA |  |
| RUNX1 |  |  | SPACA3 | RUNX1 |  |
| SPRY4-IT1 |  |  | TAF7L | SPRY4-IT1 |  |
| MIR451A |  |  | AKAP4 | MIR451A |  |
| LARS1 |  |  | CASC3 | LARS1 |  |
| NRG1 |  |  | PRAME | NRG1 |  |
| GGT1 |  |  | LZTS1 | GGT1 |  |
| BLM |  |  | CABYR | BLM |  |
| CD274 |  |  | ARX | CD274 |  |
| BARD1 |  |  | CEP290 | BARD1 |  |
| LOC126806659 |  |  | CTNNA2 | LOC126806659 |  |
| MIR152 |  |  | DLC1 | MIR152 |  |
| EGF |  |  | PIWIL2 | EGF |  |
| RINT1 |  |  | ERG | RINT1 |  |
| MIR26B |  |  | KDM5B | MIR26B |  |
| PCAT1 |  |  | CTCFL | PCAT1 |  |
| CDKN3 |  |  | FGF4 | CDKN3 |  |
| MIRLET7G |  |  | MAEL | MIRLET7G |  |
| SDHA |  |  | ODF2 | SDHA |  |
| ZFAS1 |  |  | SYTL2 | ZFAS1 |  |
| ADIPOQ |  |  | BCAR1 | ADIPOQ |  |
| SLCO1B1 |  |  | MSH2 | SLCO1B1 |  |
| IL21R |  |  |  | IL21R |  |
| MIR215 |  |  |  | MIR215 |  |
| PBRM1 |  |  |  | PBRM1 |  |
| BMPR1A |  |  |  | BMPR1A |  |
| HNF1A-AS1 |  |  |  | HNF1A-AS1 |  |
| PHKG2 |  |  |  | PHKG2 |  |
| TET2 |  |  |  | TET2 |  |
| MIR19A |  |  |  | MIR19A |  |
| CCAT2 |  |  |  | CCAT2 |  |
| CD44 |  |  |  | CD44 |  |
| CFTR |  |  |  | CFTR |  |
| PIK3CG |  |  |  | PIK3CG |  |
| FLT3 |  |  |  | FLT3 |  |
| MMP9 |  |  |  | MMP9 |  |
| PMS1 |  |  |  | PMS1 |  |
| SLC2A2 |  |  |  | SLC2A2 |  |
| POLE |  |  |  | POLE |  |
| PPARA |  |  |  | PPARA |  |
| MIR199B |  |  |  | MIR199B |  |
| MIR96 |  |  |  | MIR96 |  |
| AURKA |  |  |  | AURKA |  |
| MIR185 |  |  |  | MIR185 |  |
| MIR338 |  |  |  | MIR338 |  |
| CRNDE |  |  |  | CRNDE |  |
| MIR181B1 |  |  |  | MIR181B1 |  |
| POLD1 |  |  |  | POLD1 |  |
| MMP2 |  |  |  | MMP2 |  |
| CBL |  |  |  | CBL |  |
| MIRLET7E |  |  |  | MIRLET7E |  |
| ARG1 |  |  |  | ARG1 |  |
| FABP1 |  |  |  | FABP1 |  |
| AFAP1-AS1 |  |  |  | AFAP1-AS1 |  |
| CXCL8 |  |  |  | CXCL8 |  |
| MIR324 |  |  |  | MIR324 |  |
| ERCC2 |  |  |  | ERCC2 |  |
| ITGB1 |  |  |  | ITGB1 |  |
| MKI67 |  |  |  | MKI67 |  |
| GFER |  |  |  | GFER |  |
| HOXA11-AS |  |  |  | HOXA11-AS |  |
| FAH |  |  |  | FAH |  |
| WRN |  |  |  | WRN |  |
| RAD51C |  |  |  | RAD51C |  |
| AXIN2 |  |  |  | AXIN2 |  |
| VIM |  |  |  | VIM |  |
| HADHA |  |  |  | HADHA |  |
| CDC25A |  |  |  | CDC25A |  |
| AXL |  |  |  | AXL |  |
| NR1H3 |  |  |  | NR1H3 |  |
| ATRX |  |  |  | ATRX |  |
| APOB |  |  |  | APOB |  |
| IL12RB1 |  |  |  | IL12RB1 |  |
| WRAP53 |  |  |  | WRAP53 |  |
| MIR301A |  |  |  | MIR301A |  |
| MIR146B |  |  |  | MIR146B |  |
| CP |  |  |  | CP |  |
| SLC25A13 |  |  |  | SLC25A13 |  |
| MPL |  |  |  | MPL |  |
| CCL2 |  |  |  | CCL2 |  |
| MECOM |  |  |  | MECOM |  |
| MIRLET7A1 |  |  |  | MIRLET7A1 |  |
| SNHG1 |  |  |  | SNHG1 |  |
| MIR216A |  |  |  | MIR216A |  |
| BANCR |  |  |  | BANCR |  |
| MIR16-1 |  |  |  | MIR16-1 |  |
| PTGS2 |  |  |  | PTGS2 |  |
| HAGLR |  |  |  | HAGLR |  |
| NPM1 |  |  |  | NPM1 |  |
| CPT1A |  |  |  | CPT1A |  |
| POLG |  |  |  | POLG |  |
| AMACR |  |  |  | AMACR |  |
| DNMT3A |  |  |  | DNMT3A |  |
| SLC2A1 |  |  |  | SLC2A1 |  |
| ROS1 |  |  |  | ROS1 |  |
| CYP1A2 |  |  |  | CYP1A2 |  |
| ACE |  |  |  | ACE |  |
| PFKFB1 |  |  |  | PFKFB1 |  |
| IFNA1 |  |  |  | IFNA1 |  |
| MIR210 |  |  |  | MIR210 |  |
| IGF1R |  |  |  | IGF1R |  |
| LRP6 |  |  |  | LRP6 |  |
| ABL1 |  |  |  | ABL1 |  |
| PANDAR |  |  |  | PANDAR |  |
| MTHFR |  |  |  | MTHFR |  |
| MIR27B |  |  |  | MIR27B |  |
| STAT1 |  |  |  | STAT1 |  |
| DNMT1 |  |  |  | DNMT1 |  |
| NKX2-1 |  |  |  | NKX2-1 |  |
| MSR1 |  |  |  | MSR1 |  |
| AKT2 |  |  |  | AKT2 |  |
| APOE |  |  |  | APOE |  |
| GATA3 |  |  |  | GATA3 |  |
| GSTM1 |  |  |  | GSTM1 |  |
| KRT8 |  |  |  | KRT8 |  |
| DDR2 |  |  |  | DDR2 |  |
| CYP1A1 |  |  |  | CYP1A1 |  |
| LEP |  |  |  | LEP |  |
| PTH |  |  |  | PTH |  |
| C11orf65 |  |  |  | C11orf65 |  |
| MGMT |  |  |  | MGMT |  |
| LOC110806263 |  |  |  | LOC110806263 |  |
| PKLR |  |  |  | PKLR |  |
| MIR181C |  |  |  | MIR181C |  |
| JAK1 |  |  |  | JAK1 |  |
| CDKN1C |  |  |  | CDKN1C |  |
| RHOA |  |  |  | RHOA |  |
| FANCD2 |  |  |  | FANCD2 |  |
| SETD2 |  |  |  | SETD2 |  |
| PCNA |  |  |  | PCNA |  |
| PTPRC |  |  |  | PTPRC |  |
| FGFR4 |  |  |  | FGFR4 |  |
| TP53COR1 |  |  |  | TP53COR1 |  |
| GREM1 |  |  |  | GREM1 |  |
| MIR128-2 |  |  |  | MIR128-2 |  |
| XBP1 |  |  |  | XBP1 |  |
| RELA |  |  |  | RELA |  |
| RAD51 |  |  |  | RAD51 |  |
| FBXW7 |  |  |  | FBXW7 |  |
| MIR181A2 |  |  |  | MIR181A2 |  |
| GATA2 |  |  |  | GATA2 |  |
| NR1H2 |  |  |  | NR1H2 |  |
| PDGFRL |  |  |  | PDGFRL |  |
| MIR186 |  |  |  | MIR186 |  |
| MYCN |  |  |  | MYCN |  |
| DGCR5 |  |  |  | DGCR5 |  |
| SNHG12 |  |  |  | SNHG12 |  |
| MLH3 |  |  |  | MLH3 |  |
| CAV1 |  |  |  | CAV1 |  |
| MIR199A1 |  |  |  | MIR199A1 |  |
| SOX2-OT |  |  |  | SOX2-OT |  |
| WT1-AS |  |  |  | WT1-AS |  |
| TERC |  |  |  | TERC |  |
| LIG4 |  |  |  | LIG4 |  |
| EWSR1 |  |  |  | EWSR1 |  |
| FANCC |  |  |  | FANCC |  |
| SMAD2 |  |  |  | SMAD2 |  |
| FANCA |  |  |  | FANCA |  |
| SOD2 |  |  |  | SOD2 |  |
| B2M |  |  |  | B2M |  |
| NPTN-IT1 |  |  |  | NPTN-IT1 |  |
| FOXO1 |  |  |  | FOXO1 |  |
| BMP6 |  |  |  | BMP6 |  |
| LIPA |  |  |  | LIPA |  |
| CCNE1 |  |  |  | CCNE1 |  |
| HAMP |  |  |  | HAMP |  |
| TFDP1 |  |  |  | TFDP1 |  |
| AGT |  |  |  | AGT |  |
| MIR148B |  |  |  | MIR148B |  |
| MRE11 |  |  |  | MRE11 |  |
| SNHG6 |  |  |  | SNHG6 |  |
| NOS2 |  |  |  | NOS2 |  |
| FBN1 |  |  |  | FBN1 |  |
| ALG9 |  |  |  | ALG9 |  |
| CDK6 |  |  |  | CDK6 |  |
| ASXL1 |  |  |  | ASXL1 |  |
| NORAD |  |  |  | NORAD |  |
| GCK |  |  |  | GCK |  |
| LIVAR |  |  |  | LIVAR |  |
| SF3B2 |  |  |  | SF3B2 |  |
| SRA1 |  |  |  | SRA1 |  |
| SMAD3 |  |  |  | SMAD3 |  |
| BIRC5 |  |  |  | BIRC5 |  |
| SNHG15 |  |  |  | SNHG15 |  |
| ETV6 |  |  |  | ETV6 |  |
| SLCO1B3 |  |  |  | SLCO1B3 |  |
| IGF2-AS |  |  |  | IGF2-AS |  |
| TMEM67 |  |  |  | TMEM67 |  |
| CASP3 |  |  |  | CASP3 |  |
| STARD13 |  |  |  | STARD13 |  |
| PFKL |  |  |  | PFKL |  |
| MIR374A |  |  |  | MIR374A |  |
| HEIH |  |  |  | HEIH |  |
| CBR3-AS1 |  |  |  | CBR3-AS1 |  |
| TF |  |  |  | TF |  |
| ACTC1 |  |  |  | ACTC1 |  |
| CASC15 |  |  |  | CASC15 |  |
| MIR30C1 |  |  |  | MIR30C1 |  |
| ABCA1 |  |  |  | ABCA1 |  |
| HLA-DRB1 |  |  |  | HLA-DRB1 |  |
| CD34 |  |  |  | CD34 |  |
| ALDH2 |  |  |  | ALDH2 |  |
| EGFR-AS1 |  |  |  | EGFR-AS1 |  |
| RECQL4 |  |  |  | RECQL4 |  |
| SST |  |  |  | SST |  |
| MIR378A |  |  |  | MIR378A |  |
| GSTP1 |  |  |  | GSTP1 |  |
| MIR424 |  |  |  | MIR424 |  |
| CDC73 |  |  |  | CDC73 |  |
| MYD88 |  |  |  | MYD88 |  |
| PDGFB |  |  |  | PDGFB |  |
| APOA1 |  |  |  | APOA1 |  |
| LNCRNA-ATB |  |  |  | LNCRNA-ATB |  |
| MIR34C |  |  |  | MIR34C |  |
| MIR24-2 |  |  |  | MIR24-2 |  |
| LINC00261 |  |  |  | LINC00261 |  |
| MIR22HG |  |  |  | MIR22HG |  |
| ERCC4 |  |  |  | ERCC4 |  |
| RAD51D |  |  |  | RAD51D |  |
| FANCM |  |  |  | FANCM |  |
| CHEK1 |  |  |  | CHEK1 |  |
| CEACAM5 |  |  |  | CEACAM5 |  |
| ALDOB |  |  |  | ALDOB |  |
| TLR4 |  |  |  | TLR4 |  |
| IRS1 |  |  |  | IRS1 |  |
| FOXP3 |  |  |  | FOXP3 |  |
| IL7R |  |  |  | IL7R |  |
| GLI1 |  |  |  | GLI1 |  |
| TIMP1 |  |  |  | TIMP1 |  |
| SUFU |  |  |  | SUFU |  |
| TYMS |  |  |  | TYMS |  |
| ALPL |  |  |  | ALPL |  |
| MIR136 |  |  |  | MIR136 |  |
| OFD1 |  |  |  | OFD1 |  |
| LINC01672 |  |  |  | LINC01672 |  |
| MIR494 |  |  |  | MIR494 |  |
| MIR196A2 |  |  |  | MIR196A2 |  |
| ATR |  |  |  | ATR |  |
| NNT-AS1 |  |  |  | NNT-AS1 |  |
| FALEC |  |  |  | FALEC |  |
| PDCD1 |  |  |  | PDCD1 |  |
| ATP8B1 |  |  |  | ATP8B1 |  |
| MME |  |  |  | MME |  |
| MIR483 |  |  |  | MIR483 |  |
| CASR |  |  |  | CASR |  |
| MIR429 |  |  |  | MIR429 |  |
| MUC16 |  |  |  | MUC16 |  |
| PGR |  |  |  | PGR |  |
| SLC5A5 |  |  |  | SLC5A5 |  |
| MIR335 |  |  |  | MIR335 |  |
| RAB4B-EGLN2 |  |  |  | RAB4B-EGLN2 |  |
| FLT1 |  |  |  | FLT1 |  |
| LOC126862571 |  |  |  | LOC126862571 |  |
| MSH3 |  |  |  | MSH3 |  |
| CAMTA1 |  |  |  | CAMTA1 |  |
| FANCI |  |  |  | FANCI |  |
| ALPP |  |  |  | ALPP |  |
| CD79A |  |  |  | CD79A |  |
| PAX5 |  |  |  | PAX5 |  |
| HOXA-AS2 |  |  |  | HOXA-AS2 |  |
| SERPINE1 |  |  |  | SERPINE1 |  |
| BTK |  |  |  | BTK |  |
| HMOX1 |  |  |  | HMOX1 |  |
| SREBF1 |  |  |  | SREBF1 |  |
| TCF7 |  |  |  | TCF7 |  |
| MIR373 |  |  |  | MIR373 |  |
| DPYD |  |  |  | DPYD |  |
| CYP7A1 |  |  |  | CYP7A1 |  |
| CDK2 |  |  |  | CDK2 |  |
| TUSC7 |  |  |  | TUSC7 |  |
| PTENP1 |  |  |  | PTENP1 |  |
| GHET1 |  |  |  | GHET1 |  |
| ZEB1-AS1 |  |  |  | ZEB1-AS1 |  |
| PAX8 |  |  |  | PAX8 |  |
| TTR |  |  |  | TTR |  |
| SPP1 |  |  |  | SPP1 |  |
| BCL6 |  |  |  | BCL6 |  |
| MAPK8 |  |  |  | MAPK8 |  |
| MIR33A |  |  |  | MIR33A |  |
| EPAS1 |  |  |  | EPAS1 |  |
| DKK3 |  |  |  | DKK3 |  |
| SOCS1 |  |  |  | SOCS1 |  |
| MUC2 |  |  |  | MUC2 |  |
| CERNA2 |  |  |  | CERNA2 |  |
| BUB1B |  |  |  | BUB1B |  |
| CSF3 |  |  |  | CSF3 |  |
| BCR |  |  |  | BCR |  |
| ZEB2-AS1 |  |  |  | ZEB2-AS1 |  |
| SNHG20 |  |  |  | SNHG20 |  |
| NR3C1 |  |  |  | NR3C1 |  |
| KMT2D |  |  |  | KMT2D |  |
| SMAD7 |  |  |  | SMAD7 |  |
| TJP2 |  |  |  | TJP2 |  |
| CEP290 |  |  |  | CEP290 |  |
| PKD1-AS1 |  |  |  | PKD1-AS1 |  |
| SLC37A4 |  |  |  | SLC37A4 |  |
| FGF2 |  |  |  | FGF2 |  |
| MIR219A1 |  |  |  | MIR219A1 |  |
| F3 |  |  |  | F3 |  |
| LMNA |  |  |  | LMNA |  |
| TNFRSF1A |  |  |  | TNFRSF1A |  |
| MIR192 |  |  |  | MIR192 |  |
| SOCS3 |  |  |  | SOCS3 |  |
| REN |  |  |  | REN |  |
| ABCG2 |  |  |  | ABCG2 |  |
| NFKBIA |  |  |  | NFKBIA |  |
| DNAJB11 |  |  |  | DNAJB11 |  |
| TLR2 |  |  |  | TLR2 |  |
| MITF |  |  |  | MITF |  |
| GATA1 |  |  |  | GATA1 |  |
| CYP2A6 |  |  |  | CYP2A6 |  |
| FOS |  |  |  | FOS |  |
| MIR137 |  |  |  | MIR137 |  |
| EPO |  |  |  | EPO |  |
| CYP17A1 |  |  |  | CYP17A1 |  |
| CCEPR |  |  |  | CCEPR |  |
| IL1RN |  |  |  | IL1RN |  |
| DLC1 |  |  |  | DLC1 |  |
| IKZF1 |  |  |  | IKZF1 |  |
| GSK3B |  |  |  | GSK3B |  |
| RIOX2 |  |  |  | RIOX2 |  |
| PHKB |  |  |  | PHKB |  |
| IL2RA |  |  |  | IL2RA |  |
| MIRLET7A3 |  |  |  | MIRLET7A3 |  |
| MIR500A |  |  |  | MIR500A |  |
| UGT1A9 |  |  |  | UGT1A9 |  |
| CXCL12 |  |  |  | CXCL12 |  |
| MIR100HG |  |  |  | MIR100HG |  |
| IL1A |  |  |  | IL1A |  |
| PRKDC |  |  |  | PRKDC |  |
| DRAIC |  |  |  | DRAIC |  |
| DES |  |  |  | DES |  |
| NEK8 |  |  |  | NEK8 |  |
| SYP |  |  |  | SYP |  |
| MIR103A2 |  |  |  | MIR103A2 |  |
| CDH17 |  |  |  | CDH17 |  |
| MCL1 |  |  |  | MCL1 |  |
| KMT2A |  |  |  | KMT2A |  |
| ASS1 |  |  |  | ASS1 |  |
| FOXA1 |  |  |  | FOXA1 |  |
| CYP2D6 |  |  |  | CYP2D6 |  |
| AKT3 |  |  |  | AKT3 |  |
| TYMP |  |  |  | TYMP |  |
| CYP2C19 |  |  |  | CYP2C19 |  |
| MED12 |  |  |  | MED12 |  |
| AIP |  |  |  | AIP |  |
| MMUT |  |  |  | MMUT |  |
| FHIT |  |  |  | FHIT |  |
| PC |  |  |  | PC |  |
| MIR106A |  |  |  | MIR106A |  |
| NPC1 |  |  |  | NPC1 |  |
| DNMT3B |  |  |  | DNMT3B |  |
| MIR296 |  |  |  | MIR296 |  |
| CYCS |  |  |  | CYCS |  |
| XIAP |  |  |  | XIAP |  |
| CTNNA1 |  |  |  | CTNNA1 |  |
| PCAT29 |  |  |  | PCAT29 |  |
| MIR125B2 |  |  |  | MIR125B2 |  |
| SMPD1 |  |  |  | SMPD1 |  |
| LDLR |  |  |  | LDLR |  |
| PCBP2-OT1 |  |  |  | PCBP2-OT1 |  |
| GFOD3P |  |  |  | GFOD3P |  |
| LINC00941 |  |  |  | LINC00941 |  |
| FARSB |  |  |  | FARSB |  |
| CERNA3 |  |  |  | CERNA3 |  |
| CD36 |  |  |  | CD36 |  |
| FER1L4 |  |  |  | FER1L4 |  |
| TGFBR1 |  |  |  | TGFBR1 |  |
| MIR133B |  |  |  | MIR133B |  |
| BCL2L1 |  |  |  | BCL2L1 |  |
| ACTB |  |  |  | ACTB |  |
| GBA1 |  |  |  | GBA1 |  |
| NR2F1-AS1 |  |  |  | NR2F1-AS1 |  |
| ABCC1 |  |  |  | ABCC1 |  |
| LEPR |  |  |  | LEPR |  |
| IRS2 |  |  |  | IRS2 |  |
| PRF1 |  |  |  | PRF1 |  |
| FN1 |  |  |  | FN1 |  |
| POLR1HASP |  |  |  | POLR1HASP |  |
| MAX |  |  |  | MAX |  |
| JPX |  |  |  | JPX |  |
| NTHL1 |  |  |  | NTHL1 |  |
| THADA |  |  |  | THADA |  |
| MMP1 |  |  |  | MMP1 |  |
| TG |  |  |  | TG |  |
| MIR320A |  |  |  | MIR320A |  |
| CHGA |  |  |  | CHGA |  |
| CYP19A1 |  |  |  | CYP19A1 |  |
| CD4 |  |  |  | CD4 |  |
| GNAQ |  |  |  | GNAQ |  |
| F5 |  |  |  | F5 |  |
| MTUS1 |  |  |  | MTUS1 |  |
| IL4 |  |  |  | IL4 |  |
| VLDLR-AS1 |  |  |  | VLDLR-AS1 |  |
| NPHP1 |  |  |  | NPHP1 |  |
| RPGRIP1L |  |  |  | RPGRIP1L |  |
| ADA |  |  |  | ADA |  |
| IL17A |  |  |  | IL17A |  |
| TNFSF10 |  |  |  | TNFSF10 |  |
| TGFA |  |  |  | TGFA |  |
| FECH |  |  |  | FECH |  |
| IGFBP3 |  |  |  | IGFBP3 |  |
| TP63 |  |  |  | TP63 |  |
| PRKCD |  |  |  | PRKCD |  |
| SIRT1 |  |  |  | SIRT1 |  |
| SDHAF2 |  |  |  | SDHAF2 |  |
| MPV17 |  |  |  | MPV17 |  |
| MIR100 |  |  |  | MIR100 |  |
| MT1DP |  |  |  | MT1DP |  |
| PRECSIT |  |  |  | PRECSIT |  |
| CPS1-IT1 |  |  |  | CPS1-IT1 |  |
| MIR204 |  |  |  | MIR204 |  |
| CEACAM3 |  |  |  | CEACAM3 |  |
| NPHP3 |  |  |  | NPHP3 |  |
| OTC |  |  |  | OTC |  |
| LNCBRM |  |  |  | LNCBRM |  |
| RUVBL1 |  |  |  | RUVBL1 |  |
| HOXA13 |  |  |  | HOXA13 |  |
| NME1 |  |  |  | NME1 |  |
| ABCC3 |  |  |  | ABCC3 |  |
| MAP2K2 |  |  |  | MAP2K2 |  |
| CLDN1 |  |  |  | CLDN1 |  |
| LINC00665 |  |  |  | LINC00665 |  |
| CAT |  |  |  | CAT |  |
| DCDC2 |  |  |  | DCDC2 |  |
| NKILA |  |  |  | NKILA |  |
| MIR98 |  |  |  | MIR98 |  |
| STAT5B |  |  |  | STAT5B |  |
| MIR124-2 |  |  |  | MIR124-2 |  |
| JAK3 |  |  |  | JAK3 |  |
| FANCL |  |  |  | FANCL |  |
| MIR149 |  |  |  | MIR149 |  |
| RMRP |  |  |  | RMRP |  |
| FANCF |  |  |  | FANCF |  |
| MIRLET7F2 |  |  |  | MIRLET7F2 |  |
| MIR15B |  |  |  | MIR15B |  |
| FLT4 |  |  |  | FLT4 |  |
| ERCC1 |  |  |  | ERCC1 |  |
| PARP1 |  |  |  | PARP1 |  |
| SETBP1 |  |  |  | SETBP1 |  |
| RARB |  |  |  | RARB |  |
| MAP3K20 |  |  |  | MAP3K20 |  |
| RASSF1 |  |  |  | RASSF1 |  |
| LINC01419 |  |  |  | LINC01419 |  |
| MIR361 |  |  |  | MIR361 |  |
| STEAP4 |  |  |  | STEAP4 |  |
| NR1I2 |  |  |  | NR1I2 |  |
| MIR181B2 |  |  |  | MIR181B2 |  |
| SHOC2 |  |  |  | SHOC2 |  |
| KLF6 |  |  |  | KLF6 |  |
| MIR10A |  |  |  | MIR10A |  |
| PLAU |  |  |  | PLAU |  |
| CC2D2A |  |  |  | CC2D2A |  |
| LZTR1 |  |  |  | LZTR1 |  |
| HDAC1 |  |  |  | HDAC1 |  |
| MCU |  |  |  | MCU |  |
| PRKACA |  |  |  | PRKACA |  |
| SEC61A1 |  |  |  | SEC61A1 |  |
| NOS3 |  |  |  | NOS3 |  |
| SOS1 |  |  |  | SOS1 |  |
| SOX9 |  |  |  | SOX9 |  |
| HSD17B13 |  |  |  | HSD17B13 |  |
| IL6R |  |  |  | IL6R |  |
| SH2B3 |  |  |  | SH2B3 |  |
| MIR30D |  |  |  | MIR30D |  |
| PTK2 |  |  |  | PTK2 |  |
| TCF4 |  |  |  | TCF4 |  |
| ENO2 |  |  |  | ENO2 |  |
| LGALS3 |  |  |  | LGALS3 |  |
| DBH-AS1 |  |  |  | DBH-AS1 |  |
| NPHP4 |  |  |  | NPHP4 |  |
| SOD2-OT1 |  |  |  | SOD2-OT1 |  |
| FBP1 |  |  |  | FBP1 |  |
| HSPA6 |  |  |  | HSPA6 |  |
| FANCE |  |  |  | FANCE |  |
| MIR9-2 |  |  |  | MIR9-2 |  |
| MT-CYB |  |  |  | MT-CYB |  |
| PCSK9 |  |  |  | PCSK9 |  |
| MMP7 |  |  |  | MMP7 |  |
| LINC00589 |  |  |  | LINC00589 |  |
| TFR2 |  |  |  | TFR2 |  |
| TOP2A |  |  |  | TOP2A |  |
| NSD1 |  |  |  | NSD1 |  |
| TMEM127 |  |  |  | TMEM127 |  |
| GSTT1 |  |  |  | GSTT1 |  |
| CDKN2C |  |  |  | CDKN2C |  |
| GJA1 |  |  |  | GJA1 |  |
| CASC11 |  |  |  | CASC11 |  |
| SOX2 |  |  |  | SOX2 |  |
| SOD1 |  |  |  | SOD1 |  |
| FASN |  |  |  | FASN |  |
| SERPINC1 |  |  |  | SERPINC1 |  |
| HBB |  |  |  | HBB |  |
| MIR24-1 |  |  |  | MIR24-1 |  |
| YARS1 |  |  |  | YARS1 |  |
| SRSF2 |  |  |  | SRSF2 |  |
| TIMP2 |  |  |  | TIMP2 |  |
| GFAP |  |  |  | GFAP |  |
| TTF1 |  |  |  | TTF1 |  |
| CNOT9 |  |  |  | CNOT9 |  |
| FOCAD |  |  |  | FOCAD |  |
| TNFRSF10B |  |  |  | TNFRSF10B |  |
| ACTA2 |  |  |  | ACTA2 |  |
| LINC01138 |  |  |  | LINC01138 |  |
| BAAT |  |  |  | BAAT |  |
| FTX |  |  |  | FTX |  |
| LINC01018 |  |  |  | LINC01018 |  |
| DGUOK |  |  |  | DGUOK |  |
| IFNA2 |  |  |  | IFNA2 |  |
| LINC02605 |  |  |  | LINC02605 |  |
| RARA |  |  |  | RARA |  |
| MIRLET7A2 |  |  |  | MIRLET7A2 |  |
| NQO1 |  |  |  | NQO1 |  |
| KEAP1 |  |  |  | KEAP1 |  |
| AGXT |  |  |  | AGXT |  |
| AOC4P |  |  |  | AOC4P |  |
| CARD8-AS1 |  |  |  | CARD8-AS1 |  |
| MIR95 |  |  |  | MIR95 |  |
| CES1 |  |  |  | CES1 |  |
| CYP2C9 |  |  |  | CYP2C9 |  |
| RNF43 |  |  |  | RNF43 |  |
| LINC00926 |  |  |  | LINC00926 |  |
| PRPSAP1 |  |  |  | PRPSAP1 |  |
| ACTA2-AS1 |  |  |  | ACTA2-AS1 |  |
| UMOD |  |  |  | UMOD |  |
| BMP7 |  |  |  | BMP7 |  |
| CCND3 |  |  |  | CCND3 |  |
| LYN |  |  |  | LYN |  |
| G6PD |  |  |  | G6PD |  |
| SOX10 |  |  |  | SOX10 |  |
| STAT5A |  |  |  | STAT5A |  |
| KRTAP5-AS1 |  |  |  | KRTAP5-AS1 |  |
| KCTD13 |  |  |  | KCTD13 |  |
| AHR |  |  |  | AHR |  |
| SNHG3 |  |  |  | SNHG3 |  |
| HSP90AA1 |  |  |  | HSP90AA1 |  |
| PCNA-AS1 |  |  |  | PCNA-AS1 |  |
| MPO |  |  |  | MPO |  |
| PIK3CD |  |  |  | PIK3CD |  |
| UGT1A7 |  |  |  | UGT1A7 |  |
| MIR26A2 |  |  |  | MIR26A2 |  |
| CSF1R |  |  |  | CSF1R |  |
| CD40LG |  |  |  | CD40LG |  |
| ONECUT1 |  |  |  | ONECUT1 |  |
| CD8A |  |  |  | CD8A |  |
| LOC129933707 |  |  |  | LOC129933707 |  |
| FANCG |  |  |  | FANCG |  |
| PRKN |  |  |  | PRKN |  |
| S100B |  |  |  | S100B |  |
| EDN1 |  |  |  | EDN1 |  |
| GNA11 |  |  |  | GNA11 |  |
| POT1 |  |  |  | POT1 |  |
| MTTP |  |  |  | MTTP |  |
| ERCC5 |  |  |  | ERCC5 |  |
| DDIT3 |  |  |  | DDIT3 |  |
| CREB1 |  |  |  | CREB1 |  |
| PRKCA |  |  |  | PRKCA |  |
| CDH4 |  |  |  | CDH4 |  |
| MIR9-1 |  |  |  | MIR9-1 |  |
| SHH |  |  |  | SHH |  |
| XPC |  |  |  | XPC |  |
| PRAL |  |  |  | PRAL |  |
| XRCC3 |  |  |  | XRCC3 |  |
| URB2 |  |  |  | URB2 |  |
| LOC129997612 |  |  |  | LOC129997612 |  |
| HP |  |  |  | HP |  |
| TNFSF11 |  |  |  | TNFSF11 |  |
| THPO |  |  |  | THPO |  |
| MIR1-2 |  |  |  | MIR1-2 |  |
| CYP27A1 |  |  |  | CYP27A1 |  |
| TFE3 |  |  |  | TFE3 |  |
| KDM6A |  |  |  | KDM6A |  |
| XRCC2 |  |  |  | XRCC2 |  |
| MVK |  |  |  | MVK |  |
| SMARCE1 |  |  |  | SMARCE1 |  |
| GATA4 |  |  |  | GATA4 |  |
| NCAM1 |  |  |  | NCAM1 |  |
| INVS |  |  |  | INVS |  |
| POU5F1 |  |  |  | POU5F1 |  |
| DHCR7 |  |  |  | DHCR7 |  |
| C20orf204 |  |  |  | C20orf204 |  |
| FABP5P3 |  |  |  | FABP5P3 |  |
| KITLG |  |  |  | KITLG |  |
| CDH2 |  |  |  | CDH2 |  |
| GLS2 |  |  |  | GLS2 |  |
| PLG |  |  |  | PLG |  |
| HSD3B7 |  |  |  | HSD3B7 |  |
| HMGCR |  |  |  | HMGCR |  |
| LINC00173 |  |  |  | LINC00173 |  |
| MAPK3 |  |  |  | MAPK3 |  |
| PROM1 |  |  |  | PROM1 |  |
| UFC1 |  |  |  | UFC1 |  |
| HLA-B |  |  |  | HLA-B |  |
| SNHG16 |  |  |  | SNHG16 |  |
| SLC10A1 |  |  |  | SLC10A1 |  |
| SERPINA3 |  |  |  | SERPINA3 |  |
| LINC00974 |  |  |  | LINC00974 |  |
| PPIG |  |  |  | PPIG |  |
| CYP2B6 |  |  |  | CYP2B6 |  |
| CDR1 |  |  |  | CDR1 |  |
| PDIA3P1 |  |  |  | PDIA3P1 |  |
| LINC00554 |  |  |  | LINC00554 |  |
| LPL |  |  |  | LPL |  |
| MYB |  |  |  | MYB |  |
| ATG7 |  |  |  | ATG7 |  |
| BIRC3 |  |  |  | BIRC3 |  |
| RPS20 |  |  |  | RPS20 |  |
| SLC7A6 |  |  |  | SLC7A6 |  |
| VCAM1 |  |  |  | VCAM1 |  |
| ARC |  |  |  | ARC |  |
| TCF3 |  |  |  | TCF3 |  |
| TMX2-CTNND1 |  |  |  | TMX2-CTNND1 |  |
| TKT |  |  |  | TKT |  |
| MIR375 |  |  |  | MIR375 |  |
| HSD17B4 |  |  |  | HSD17B4 |  |
| BCL10 |  |  |  | BCL10 |  |
| ITCH |  |  |  | ITCH |  |
| ERCC3 |  |  |  | ERCC3 |  |
| IRF1 |  |  |  | IRF1 |  |
| PCK1 |  |  |  | PCK1 |  |
| AGL |  |  |  | AGL |  |
| MAP3K1 |  |  |  | MAP3K1 |  |
| CECR7 |  |  |  | CECR7 |  |
| XPA |  |  |  | XPA |  |
| GLT1D1 |  |  |  | GLT1D1 |  |
| GUSB |  |  |  | GUSB |  |
| NLRP3 |  |  |  | NLRP3 |  |
| ZNF674-AS1 |  |  |  | ZNF674-AS1 |  |
| LINC00601 |  |  |  | LINC00601 |  |
| TGFB2 |  |  |  | TGFB2 |  |
| FAM83A-AS1 |  |  |  | FAM83A-AS1 |  |
| CYP7B1 |  |  |  | CYP7B1 |  |
| GPC3-AS1 |  |  |  | GPC3-AS1 |  |
| EGILA |  |  |  | EGILA |  |
| IATPR |  |  |  | IATPR |  |
| POMC |  |  |  | POMC |  |
| MKS1 |  |  |  | MKS1 |  |
| CDX2 |  |  |  | CDX2 |  |
| RBP4 |  |  |  | RBP4 |  |
| NR5A2 |  |  |  | NR5A2 |  |
| BAIAP2-DT |  |  |  | BAIAP2-DT |  |
| MIRLET7I |  |  |  | MIRLET7I |  |
| OVCH1-AS1 |  |  |  | OVCH1-AS1 |  |
| HMGA2 |  |  |  | HMGA2 |  |
| RTEL1 |  |  |  | RTEL1 |  |
| AGTR1 |  |  |  | AGTR1 |  |
| CD19 |  |  |  | CD19 |  |
| LINC02027 |  |  |  | LINC02027 |  |
| TYR |  |  |  | TYR |  |
| MMP14 |  |  |  | MMP14 |  |
| PMM2 |  |  |  | PMM2 |  |
| SEPSECS |  |  |  | SEPSECS |  |
| CCN2 |  |  |  | CCN2 |  |
| AKR1D1 |  |  |  | AKR1D1 |  |
| MAPKAPK5-AS1 |  |  |  | MAPKAPK5-AS1 |  |
| NAT2 |  |  |  | NAT2 |  |
| ERCC6 |  |  |  | ERCC6 |  |
| BCYRN1 |  |  |  | BCYRN1 |  |
| PLAUR |  |  |  | PLAUR |  |
| LINC02055 |  |  |  | LINC02055 |  |
| CD82 |  |  |  | CD82 |  |
| HSPA5 |  |  |  | HSPA5 |  |
| SLC40A1 |  |  |  | SLC40A1 |  |
| TMEM51-AS1 |  |  |  | TMEM51-AS1 |  |
| SLC25A27 |  |  |  | SLC25A27 |  |
| SEMA6A-AS1 |  |  |  | SEMA6A-AS1 |  |
| RAC1 |  |  |  | RAC1 |  |
| LDC1P |  |  |  | LDC1P |  |
| CXCL1P1 |  |  |  | CXCL1P1 |  |
| GAS5-AS1 |  |  |  | GAS5-AS1 |  |
| ZEB1 |  |  |  | ZEB1 |  |
| MAPK14 |  |  |  | MAPK14 |  |
| DHFR |  |  |  | DHFR |  |
| ANGPT2 |  |  |  | ANGPT2 |  |
| GIHCG |  |  |  | GIHCG |  |
| MIR328 |  |  |  | MIR328 |  |
| PDX1 |  |  |  | PDX1 |  |
| CDK1 |  |  |  | CDK1 |  |
| STAT6 |  |  |  | STAT6 |  |
| EXT2 |  |  |  | EXT2 |  |
| ICAM4-AS1 |  |  |  | ICAM4-AS1 |  |
| HTR2A-AS1 |  |  |  | HTR2A-AS1 |  |
| WWOX |  |  |  | WWOX |  |
| PRMT7 |  |  |  | PRMT7 |  |
| ATP7A |  |  |  | ATP7A |  |
| CPT2 |  |  |  | CPT2 |  |
| MYH11 |  |  |  | MYH11 |  |
| SCYL1 |  |  |  | SCYL1 |  |
| CYLD |  |  |  | CYLD |  |
| TSHR |  |  |  | TSHR |  |
| TCF7L2 |  |  |  | TCF7L2 |  |
| SPINK1 |  |  |  | SPINK1 |  |
| NARF-AS1 |  |  |  | NARF-AS1 |  |
| SIRT1-AS |  |  |  | SIRT1-AS |  |
| LIPC |  |  |  | LIPC |  |
| HJV |  |  |  | HJV |  |
| ZNF350-AS1 |  |  |  | ZNF350-AS1 |  |
| C14orf132 |  |  |  | C14orf132 |  |
| LINC00210 |  |  |  | LINC00210 |  |
| CCNA2 |  |  |  | CCNA2 |  |
| MIR34B |  |  |  | MIR34B |  |
| NOD2 |  |  |  | NOD2 |  |
| PRC1-AS1 |  |  |  | PRC1-AS1 |  |
| CCND3P1 |  |  |  | CCND3P1 |  |
| SP1 |  |  |  | SP1 |  |
| CA3-AS1 |  |  |  | CA3-AS1 |  |
| SHBG |  |  |  | SHBG |  |
| NOTCH3 |  |  |  | NOTCH3 |  |
| MIR154 |  |  |  | MIR154 |  |
| CCND2 |  |  |  | CCND2 |  |
| HLA-A |  |  |  | HLA-A |  |
| THY1-AS1 |  |  |  | THY1-AS1 |  |
| ULK4P2 |  |  |  | ULK4P2 |  |
| HMBS |  |  |  | HMBS |  |
| DIP2C-AS1 |  |  |  | DIP2C-AS1 |  |
| SERHL |  |  |  | SERHL |  |
| LINC00383 |  |  |  | LINC00383 |  |
| TIMP3 |  |  |  | TIMP3 |  |
| MIR197 |  |  |  | MIR197 |  |
| UROD |  |  |  | UROD |  |
| ATP4A |  |  |  | ATP4A |  |
| ELN |  |  |  | ELN |  |
| PPM1D |  |  |  | PPM1D |  |
| DNAH8 |  |  |  | DNAH8 |  |
| CXCL10 |  |  |  | CXCL10 |  |
| SNAI1 |  |  |  | SNAI1 |  |
| HLA-DQB1 |  |  |  | HLA-DQB1 |  |
| DZIP1L |  |  |  | DZIP1L |  |
| CFH |  |  |  | CFH |  |
| HOXB13 |  |  |  | HOXB13 |  |
| PRL |  |  |  | PRL |  |
| POLG2 |  |  |  | POLG2 |  |
| SQSTM1 |  |  |  | SQSTM1 |  |
| VEGFC |  |  |  | VEGFC |  |
| GBE1 |  |  |  | GBE1 |  |
| ACOX1 |  |  |  | ACOX1 |  |
| CPOX |  |  |  | CPOX |  |
| TMEM216 |  |  |  | TMEM216 |  |
| BMP2 |  |  |  | BMP2 |  |
| MBL2 |  |  |  | MBL2 |  |
| SDC1 |  |  |  | SDC1 |  |
| XDH |  |  |  | XDH |  |
| ZEB2 |  |  |  | ZEB2 |  |
| EPHB4 |  |  |  | EPHB4 |  |
| SYK |  |  |  | SYK |  |
| CDK12 |  |  |  | CDK12 |  |
| CSF2 |  |  |  | CSF2 |  |
| U2AF1 |  |  |  | U2AF1 |  |
| IFNB1 |  |  |  | IFNB1 |  |
| SHC1 |  |  |  | SHC1 |  |
| ELANE |  |  |  | ELANE |  |
| BSG |  |  |  | BSG |  |
| TWIST1 |  |  |  | TWIST1 |  |
| IGFBP1 |  |  |  | IGFBP1 |  |
| DKC1 |  |  |  | DKC1 |  |
| ESR2 |  |  |  | ESR2 |  |
| MIR187 |  |  |  | MIR187 |  |
| GJB2 |  |  |  | GJB2 |  |
| MIR30B |  |  |  | MIR30B |  |
| CYP3A5 |  |  |  | CYP3A5 |  |
| GLA |  |  |  | GLA |  |
| XRCC1 |  |  |  | XRCC1 |  |
| HMGB1 |  |  |  | HMGB1 |  |
| CCL5 |  |  |  | CCL5 |  |
| PTPN3 |  |  |  | PTPN3 |  |
| TPMT |  |  |  | TPMT |  |
| ROCK1 |  |  |  | ROCK1 |  |
| BSCL2 |  |  |  | BSCL2 |  |
| GAPDH |  |  |  | GAPDH |  |
| E2F1 |  |  |  | E2F1 |  |
| CALB2 |  |  |  | CALB2 |  |
| EPHX1 |  |  |  | EPHX1 |  |
| INPP5E |  |  |  | INPP5E |  |
| IL18 |  |  |  | IL18 |  |
| KLK3 |  |  |  | KLK3 |  |
| RAD54L |  |  |  | RAD54L |  |
| RAD51L3-RFFL |  |  |  | RAD51L3-RFFL |  |
| DPP4 |  |  |  | DPP4 |  |
| CTSD |  |  |  | CTSD |  |
| CD46 |  |  |  | CD46 |  |
| RAG2 |  |  |  | RAG2 |  |
| MIAT |  |  |  | MIAT |  |
| PRDM1 |  |  |  | PRDM1 |  |
| IKBKB |  |  |  | IKBKB |  |
| HDAC9 |  |  |  | HDAC9 |  |
| CSF3R |  |  |  | CSF3R |  |
| PSAP |  |  |  | PSAP |  |
| FADD |  |  |  | FADD |  |
| CASP9 |  |  |  | CASP9 |  |
| CEBPB |  |  |  | CEBPB |  |
| CCNB1 |  |  |  | CCNB1 |  |
| BDNF-AS |  |  |  | BDNF-AS |  |
| MYOM1 |  |  |  | MYOM1 |  |
| SUZ12 |  |  |  | SUZ12 |  |
| SNAI2 |  |  |  | SNAI2 |  |
| PPARGC1A |  |  |  | PPARGC1A |  |
| AHCY |  |  |  | AHCY |  |
| RAG1 |  |  |  | RAG1 |  |
| CASP10 |  |  |  | CASP10 |  |
| FGF19 |  |  |  | FGF19 |  |
| AKR1A1 |  |  |  | AKR1A1 |  |
| DYNC2H1 |  |  |  | DYNC2H1 |  |
| IRF4 |  |  |  | IRF4 |  |
| FOXA2 |  |  |  | FOXA2 |  |
| ABCG8 |  |  |  | ABCG8 |  |
| HSPA4 |  |  |  | HSPA4 |  |
| CTC1 |  |  |  | CTC1 |  |
| MEFV |  |  |  | MEFV |  |
| IFT172 |  |  |  | IFT172 |  |
| TEK |  |  |  | TEK |  |
| ABCG5 |  |  |  | ABCG5 |  |
| PKM |  |  |  | PKM |  |
| TNFRSF8 |  |  |  | TNFRSF8 |  |
| MIR342 |  |  |  | MIR342 |  |
| ABCC8 |  |  |  | ABCC8 |  |
| HSPB1 |  |  |  | HSPB1 |  |
| CPS1 |  |  |  | CPS1 |  |
| MIR130B |  |  |  | MIR130B |  |
| MIR32 |  |  |  | MIR32 |  |
| ALDOA |  |  |  | ALDOA |  |
| SLX4 |  |  |  | SLX4 |  |
| ASL |  |  |  | ASL |  |
| ANXA5 |  |  |  | ANXA5 |  |
| ABCC4 |  |  |  | ABCC4 |  |
| PHOX2B |  |  |  | PHOX2B |  |
| POR |  |  |  | POR |  |
| GAA |  |  |  | GAA |  |
| GH1 |  |  |  | GH1 |  |
| SAMHD1 |  |  |  | SAMHD1 |  |
| PALLD |  |  |  | PALLD |  |
| BCS1L |  |  |  | BCS1L |  |
| KIF1B |  |  |  | KIF1B |  |
| TINF2 |  |  |  | TINF2 |  |
| OGG1 |  |  |  | OGG1 |  |
| MIR331 |  |  |  | MIR331 |  |
| NAGLU |  |  |  | NAGLU |  |
| MAP2K4 |  |  |  | MAP2K4 |  |
| MIR423 |  |  |  | MIR423 |  |
| CYP1B1 |  |  |  | CYP1B1 |  |
| PTK2B |  |  |  | PTK2B |  |
| WNT1 |  |  |  | WNT1 |  |
| EIF2AK3 |  |  |  | EIF2AK3 |  |
| PEX5 |  |  |  | PEX5 |  |
| RPS6KB1 |  |  |  | RPS6KB1 |  |
| ACADVL |  |  |  | ACADVL |  |
| PNPLA2 |  |  |  | PNPLA2 |  |
| PIK3CB |  |  |  | PIK3CB |  |
| FGF3 |  |  |  | FGF3 |  |
| MIR211 |  |  |  | MIR211 |  |
| MIR101-1 |  |  |  | MIR101-1 |  |
| CEP164 |  |  |  | CEP164 |  |
| ADAR |  |  |  | ADAR |  |
| EGR1 |  |  |  | EGR1 |  |
| AGPAT2 |  |  |  | AGPAT2 |  |
| RAD51B |  |  |  | RAD51B |  |
| UGT1A4 |  |  |  | UGT1A4 |  |
| PPP2R1A |  |  |  | PPP2R1A |  |
| CD79B |  |  |  | CD79B |  |
| MIR92A1 |  |  |  | MIR92A1 |  |
| DMD |  |  |  | DMD |  |
| ADH1B |  |  |  | ADH1B |  |
| UGT1A6 |  |  |  | UGT1A6 |  |
| ACADM |  |  |  | ACADM |  |
| LDHA |  |  |  | LDHA |  |
| XPO1 |  |  |  | XPO1 |  |
| CLU |  |  |  | CLU |  |
| BRD4 |  |  |  | BRD4 |  |
| HMGCL |  |  |  | HMGCL |  |
| ACVRL1 |  |  |  | ACVRL1 |  |
| BMP4 |  |  |  | BMP4 |  |
| LCN2 |  |  |  | LCN2 |  |
| PTCH2 |  |  |  | PTCH2 |  |
| MIR376A1 |  |  |  | MIR376A1 |  |
| H3-3A |  |  |  | H3-3A |  |
| GLB1 |  |  |  | GLB1 |  |
| NPC2 |  |  |  | NPC2 |  |
| MIR345 |  |  |  | MIR345 |  |
| SGK1 |  |  |  | SGK1 |  |
| FOXO3 |  |  |  | FOXO3 |  |
| SOX4 |  |  |  | SOX4 |  |
| CEL |  |  |  | CEL |  |
| SLC30A10 |  |  |  | SLC30A10 |  |
| OCRL |  |  |  | OCRL |  |
| IL3 |  |  |  | IL3 |  |
| PAX2 |  |  |  | PAX2 |  |
| CASP1 |  |  |  | CASP1 |  |
| TMPRSS2 |  |  |  | TMPRSS2 |  |
| AURKB |  |  |  | AURKB |  |
| MST1 |  |  |  | MST1 |  |
| LOX |  |  |  | LOX |  |
| FAN1 |  |  |  | FAN1 |  |
| EYA1 |  |  |  | EYA1 |  |
| PIK3R2 |  |  |  | PIK3R2 |  |
| MIR144 |  |  |  | MIR144 |  |
| DAXX |  |  |  | DAXX |  |
| UGT1A |  |  |  | UGT1A |  |
| PCK2 |  |  |  | PCK2 |  |
| ALAD |  |  |  | ALAD |  |
| CLMAT3 |  |  |  | CLMAT3 |  |
| TTC21B |  |  |  | TTC21B |  |
| PEX2 |  |  |  | PEX2 |  |
| TP73 |  |  |  | TP73 |  |
| EPHX2 |  |  |  | EPHX2 |  |
| MYH9 |  |  |  | MYH9 |  |
| PECAM1 |  |  |  | PECAM1 |  |
| WDR19 |  |  |  | WDR19 |  |
| MIR128-1 |  |  |  | MIR128-1 |  |
| VWF |  |  |  | VWF |  |
| ABCB7 |  |  |  | ABCB7 |  |
| EPOR |  |  |  | EPOR |  |
| ENPP1 |  |  |  | ENPP1 |  |
| PEX1 |  |  |  | PEX1 |  |
| TOP1 |  |  |  | TOP1 |  |
| COMT |  |  |  | COMT |  |
| GATA6 |  |  |  | GATA6 |  |
| SOX11 |  |  |  | SOX11 |  |
| BBS4 |  |  |  | BBS4 |  |
| KRT5 |  |  |  | KRT5 |  |
| GAST |  |  |  | GAST |  |
| TNFRSF1B |  |  |  | TNFRSF1B |  |
| CSF1 |  |  |  | CSF1 |  |
| NCOR1 |  |  |  | NCOR1 |  |
| SEC23B |  |  |  | SEC23B |  |
| STAG2 |  |  |  | STAG2 |  |
| RRAS |  |  |  | RRAS |  |
| CD40 |  |  |  | CD40 |  |
| ALDH1A1 |  |  |  | ALDH1A1 |  |
| IDUA |  |  |  | IDUA |  |
| LCAT |  |  |  | LCAT |  |
| ADH1C |  |  |  | ADH1C |  |
| SH2D1A |  |  |  | SH2D1A |  |
| NEU1 |  |  |  | NEU1 |  |
| KIF12 |  |  |  | KIF12 |  |
| VTN |  |  |  | VTN |  |
| NFKB2 |  |  |  | NFKB2 |  |
| ERG |  |  |  | ERG |  |
| SSTR2 |  |  |  | SSTR2 |  |
| MIR193B |  |  |  | MIR193B |  |
| DDX3X |  |  |  | DDX3X |  |
| COL1A1 |  |  |  | COL1A1 |  |
| SNHG7 |  |  |  | SNHG7 |  |
| BCL2L11 |  |  |  | BCL2L11 |  |
| IDS |  |  |  | IDS |  |
| TM6SF2 |  |  |  | TM6SF2 |  |
| GDF15 |  |  |  | GDF15 |  |
| BDNF |  |  |  | BDNF |  |
| MIR31HG |  |  |  | MIR31HG |  |
| MIRLET7F1 |  |  |  | MIRLET7F1 |  |
| GNMT |  |  |  | GNMT |  |
| SKIC2 |  |  |  | SKIC2 |  |
| PIM2 |  |  |  | PIM2 |  |
| SLC22A18 |  |  |  | SLC22A18 |  |
| PHB1 |  |  |  | PHB1 |  |
| MIR124-1 |  |  |  | MIR124-1 |  |
| CD81 |  |  |  | CD81 |  |
| SNHG5 |  |  |  | SNHG5 |  |
| THBD |  |  |  | THBD |  |
| KMT2C |  |  |  | KMT2C |  |
| TFAM |  |  |  | TFAM |  |
| FLI1 |  |  |  | FLI1 |  |
| TFRC |  |  |  | TFRC |  |
| ARL13B |  |  |  | ARL13B |  |
| CYBB |  |  |  | CYBB |  |
| MIR497 |  |  |  | MIR497 |  |
| BBS2 |  |  |  | BBS2 |  |
| ANXA2 |  |  |  | ANXA2 |  |
| TRIM37 |  |  |  | TRIM37 |  |
| WAS |  |  |  | WAS |  |
| APOC3 |  |  |  | APOC3 |  |
| EDNRB |  |  |  | EDNRB |  |
| GLIS2 |  |  |  | GLIS2 |  |
| PDPN |  |  |  | PDPN |  |
| MTR |  |  |  | MTR |  |
| GHRL |  |  |  | GHRL |  |
| RETN |  |  |  | RETN |  |
| RNASEL |  |  |  | RNASEL |  |
| HAVCR2 |  |  |  | HAVCR2 |  |
| ID3 |  |  |  | ID3 |  |
| TNFAIP3 |  |  |  | TNFAIP3 |  |
| PGM1 |  |  |  | PGM1 |  |
| REST |  |  |  | REST |  |
| FLNA |  |  |  | FLNA |  |
| BCHE |  |  |  | BCHE |  |
| SCARB1 |  |  |  | SCARB1 |  |
| MUC6 |  |  |  | MUC6 |  |
| COL4A5 |  |  |  | COL4A5 |  |
| FTH1 |  |  |  | FTH1 |  |
| LRP1B |  |  |  | LRP1B |  |
| MIR206 |  |  |  | MIR206 |  |
| JUP |  |  |  | JUP |  |
| CBFB |  |  |  | CBFB |  |
| FUS |  |  |  | FUS |  |
| COL4A1 |  |  |  | COL4A1 |  |
| EXT1 |  |  |  | EXT1 |  |
| MUC5AC |  |  |  | MUC5AC |  |
| PML |  |  |  | PML |  |
| PRDM16 |  |  |  | PRDM16 |  |
| SDCCAG8 |  |  |  | SDCCAG8 |  |
| ARID1B |  |  |  | ARID1B |  |
| GPI |  |  |  | GPI |  |
| LOC129933155 |  |  |  | LOC129933155 |  |
| KCNJ11 |  |  |  | KCNJ11 |  |
| KLF4 |  |  |  | KLF4 |  |
| WNT5A |  |  |  | WNT5A |  |
| BCOR |  |  |  | BCOR |  |
| BBS9 |  |  |  | BBS9 |  |
| SBDS |  |  |  | SBDS |  |
| MT-TP |  |  |  | MT-TP |  |
| MIR542 |  |  |  | MIR542 |  |
| IL12A |  |  |  | IL12A |  |
| FGF23 |  |  |  | FGF23 |  |
| RUNX3 |  |  |  | RUNX3 |  |
| MIF |  |  |  | MIF |  |
| TXN |  |  |  | TXN |  |
| GDNF |  |  |  | GDNF |  |
| IL6ST |  |  |  | IL6ST |  |
| ALAS2 |  |  |  | ALAS2 |  |
| SPEN |  |  |  | SPEN |  |
| NGLY1 |  |  |  | NGLY1 |  |
| RUNX2 |  |  |  | RUNX2 |  |
| ODC1 |  |  |  | ODC1 |  |
| CD28 |  |  |  | CD28 |  |
| SOS2 |  |  |  | SOS2 |  |
| SKP2 |  |  |  | SKP2 |  |
| AOPEP |  |  |  | AOPEP |  |
| RASA2 |  |  |  | RASA2 |  |
| UROS |  |  |  | UROS |  |
| RAD21 |  |  |  | RAD21 |  |
| PLAT |  |  |  | PLAT |  |
| S100A4 |  |  |  | S100A4 |  |
| ABHD5 |  |  |  | ABHD5 |  |
| TRAF3 |  |  |  | TRAF3 |  |
| KRT14 |  |  |  | KRT14 |  |
| MIR193A |  |  |  | MIR193A |  |
| EPHB2 |  |  |  | EPHB2 |  |
| ETS1 |  |  |  | ETS1 |  |
| MIR485 |  |  |  | MIR485 |  |
| ELAVL1 |  |  |  | ELAVL1 |  |
| SLC22A5 |  |  |  | SLC22A5 |  |
| MAT1A |  |  |  | MAT1A |  |
| MAD1L1 |  |  |  | MAD1L1 |  |
| KIF7 |  |  |  | KIF7 |  |
| TP53BP1 |  |  |  | TP53BP1 |  |
| MIR181D |  |  |  | MIR181D |  |
| ATF4 |  |  |  | ATF4 |  |
| CST3 |  |  |  | CST3 |  |
| BBS1 |  |  |  | BBS1 |  |
| DLEU2 |  |  |  | DLEU2 |  |
| TAT |  |  |  | TAT |  |
| PRKD1 |  |  |  | PRKD1 |  |
| TCTN3 |  |  |  | TCTN3 |  |
| IRF8 |  |  |  | IRF8 |  |
| MMP3 |  |  |  | MMP3 |  |
| TLR3 |  |  |  | TLR3 |  |
| MIR29B2 |  |  |  | MIR29B2 |  |
| THBS1 |  |  |  | THBS1 |  |
| PEPD |  |  |  | PEPD |  |
| RECK |  |  |  | RECK |  |
| DIS3L2 |  |  |  | DIS3L2 |  |
| LNCARSR |  |  |  | LNCARSR |  |
| PLA2G2A |  |  |  | PLA2G2A |  |
| BECN1 |  |  |  | BECN1 |  |
| LEFTY2 |  |  |  | LEFTY2 |  |
| NAF1 |  |  |  | NAF1 |  |
| BBS5 |  |  |  | BBS5 |  |
| IL5 |  |  |  | IL5 |  |
| GRHPR |  |  |  | GRHPR |  |
| GHR |  |  |  | GHR |  |
| GDF2 |  |  |  | GDF2 |  |
| ACVR1B |  |  |  | ACVR1B |  |
| NHP2 |  |  |  | NHP2 |  |
| TNFRSF10A |  |  |  | TNFRSF10A |  |
| HADH |  |  |  | HADH |  |
| IL2RB |  |  |  | IL2RB |  |
| CCR5 |  |  |  | CCR5 |  |
| EDNRA |  |  |  | EDNRA |  |
| NPPA |  |  |  | NPPA |  |
| CD27 |  |  |  | CD27 |  |
| YY1 |  |  |  | YY1 |  |
| POU2AF1 |  |  |  | POU2AF1 |  |
| BBS10 |  |  |  | BBS10 |  |
| MALT1 |  |  |  | MALT1 |  |
| HBEGF |  |  |  | HBEGF |  |
| CFLAR |  |  |  | CFLAR |  |
| PEX14 |  |  |  | PEX14 |  |
| CD68 |  |  |  | CD68 |  |
| IQCB1 |  |  |  | IQCB1 |  |
| CUL3 |  |  |  | CUL3 |  |
| TMEM231 |  |  |  | TMEM231 |  |
| BBS7 |  |  |  | BBS7 |  |
| MC1R |  |  |  | MC1R |  |
| PPP2R1B |  |  |  | PPP2R1B |  |
| ANGPT1 |  |  |  | ANGPT1 |  |
| HMMR |  |  |  | HMMR |  |
| MIR498 |  |  |  | MIR498 |  |
| MIR202 |  |  |  | MIR202 |  |
| CTSB |  |  |  | CTSB |  |
| GALT |  |  |  | GALT |  |
| GNE |  |  |  | GNE |  |
| HDAC4 |  |  |  | HDAC4 |  |
| ZIC3 |  |  |  | ZIC3 |  |
| PEX19 |  |  |  | PEX19 |  |
| LINC00472 |  |  |  | LINC00472 |  |
| CCDC26 |  |  |  | CCDC26 |  |
| FTL |  |  |  | FTL |  |
| ENO1 |  |  |  | ENO1 |  |
| HPSE |  |  |  | HPSE |  |
| HPRT1 |  |  |  | HPRT1 |  |
| IL7 |  |  |  | IL7 |  |
| IL33 |  |  |  | IL33 |  |
| DOCK8 |  |  |  | DOCK8 |  |
| TYK2 |  |  |  | TYK2 |  |
| CD70 |  |  |  | CD70 |  |
| IGH |  |  |  | IGH |  |
| PEX13 |  |  |  | PEX13 |  |
| MST1R |  |  |  | MST1R |  |
| BUB1 |  |  |  | BUB1 |  |
| CXCR2 |  |  |  | CXCR2 |  |
| NEUROD1 |  |  |  | NEUROD1 |  |
| SGSH |  |  |  | SGSH |  |
| FMO3 |  |  |  | FMO3 |  |
| BCAR4 |  |  |  | BCAR4 |  |
| EIF2S1 |  |  |  | EIF2S1 |  |
| SELE |  |  |  | SELE |  |
| MXI1 |  |  |  | MXI1 |  |
| PPOX |  |  |  | PPOX |  |
| PEX11B |  |  |  | PEX11B |  |
| PDCD1LG2 |  |  |  | PDCD1LG2 |  |
| COL3A1 |  |  |  | COL3A1 |  |
| MIR196B |  |  |  | MIR196B |  |
| FGF4 |  |  |  | FGF4 |  |
| RABL3 |  |  |  | RABL3 |  |
| ANKS6 |  |  |  | ANKS6 |  |
| DKK1 |  |  |  | DKK1 |  |
| PEX6 |  |  |  | PEX6 |  |
| VPS33B |  |  |  | VPS33B |  |
| KCNQ1 |  |  |  | KCNQ1 |  |
| RECQL |  |  |  | RECQL |  |
| GLUD1 |  |  |  | GLUD1 |  |
| CKS1B |  |  |  | CKS1B |  |
| CEP120 |  |  |  | CEP120 |  |
| HPD |  |  |  | HPD |  |
| STXBP2 |  |  |  | STXBP2 |  |
| SERPINF1 |  |  |  | SERPINF1 |  |
| CHD7 |  |  |  | CHD7 |  |
| WDR35 |  |  |  | WDR35 |  |
| NRP1 |  |  |  | NRP1 |  |
| PEX26 |  |  |  | PEX26 |  |
| PEX16 |  |  |  | PEX16 |  |
| PPARD |  |  |  | PPARD |  |
| DNTT |  |  |  | DNTT |  |
| RNY3 |  |  |  | RNY3 |  |
| SCARNA5 |  |  |  | SCARNA5 |  |
| UGT1A8 |  |  |  | UGT1A8 |  |
| BBS12 |  |  |  | BBS12 |  |
| ALMS1 |  |  |  | ALMS1 |  |
| IFIH1 |  |  |  | IFIH1 |  |
| RASA1 |  |  |  | RASA1 |  |
| SLC19A1 |  |  |  | SLC19A1 |  |
| TFAP2A |  |  |  | TFAP2A |  |
| MTDH |  |  |  | MTDH |  |
| PTTG1 |  |  |  | PTTG1 |  |
| HADHB |  |  |  | HADHB |  |
| PIM1 |  |  |  | PIM1 |  |
| ARSA |  |  |  | ARSA |  |
| PEX10 |  |  |  | PEX10 |  |
| SSTR1 |  |  |  | SSTR1 |  |
| SPIB |  |  |  | SPIB |  |
| GALNS |  |  |  | GALNS |  |
| CTCF |  |  |  | CTCF |  |
| CYP24A1 |  |  |  | CYP24A1 |  |
| ILK |  |  |  | ILK |  |
| IKBKG |  |  |  | IKBKG |  |
| MYO5B |  |  |  | MYO5B |  |
| GRB2 |  |  |  | GRB2 |  |
| GOLM1 |  |  |  | GOLM1 |  |
| DSP |  |  |  | DSP |  |
| CCR7 |  |  |  | CCR7 |  |
| MIR499A |  |  |  | MIR499A |  |
| IGFBP2 |  |  |  | IGFBP2 |  |
| HABP2 |  |  |  | HABP2 |  |
| HIF1A-AS2 |  |  |  | HIF1A-AS2 |  |
| CA9 |  |  |  | CA9 |  |
| MIR103A1 |  |  |  | MIR103A1 |  |
| IRF5 |  |  |  | IRF5 |  |
| IRAK4 |  |  |  | IRAK4 |  |
| HLA-DQA1 |  |  |  | HLA-DQA1 |  |
| CPLANE1 |  |  |  | CPLANE1 |  |
| EXO1 |  |  |  | EXO1 |  |
| TLR9 |  |  |  | TLR9 |  |
| PON1 |  |  |  | PON1 |  |
| EGLN1 |  |  |  | EGLN1 |  |
| GPBAR1 |  |  |  | GPBAR1 |  |
| S100A1 |  |  |  | S100A1 |  |
| SLC25A20 |  |  |  | SLC25A20 |  |
| TRAF2 |  |  |  | TRAF2 |  |
| SKIC3 |  |  |  | SKIC3 |  |
| WNT4 |  |  |  | WNT4 |  |
| BHMT |  |  |  | BHMT |  |
| HSPD1 |  |  |  | HSPD1 |  |
| MIR503 |  |  |  | MIR503 |  |
| ADA2 |  |  |  | ADA2 |  |
| SRD5A2 |  |  |  | SRD5A2 |  |
| ELAC2 |  |  |  | ELAC2 |  |
| AIRE |  |  |  | AIRE |  |
| LAMP2 |  |  |  | LAMP2 |  |
| SEPTIN9 |  |  |  | SEPTIN9 |  |
| PRKAG2 |  |  |  | PRKAG2 |  |
| CARD11 |  |  |  | CARD11 |  |
| UGT1A10 |  |  |  | UGT1A10 |  |
| CD14 |  |  |  | CD14 |  |
| SLC7A9 |  |  |  | SLC7A9 |  |
| SCP2 |  |  |  | SCP2 |  |
| ZFPM2 |  |  |  | ZFPM2 |  |
| IL13 |  |  |  | IL13 |  |
| FGA |  |  |  | FGA |  |
| MIR377 |  |  |  | MIR377 |  |
| FGL1 |  |  |  | FGL1 |  |
| CXCL9 |  |  |  | CXCL9 |  |
| GJB1 |  |  |  | GJB1 |  |
| ABRAXAS1 |  |  |  | ABRAXAS1 |  |
| TAL1 |  |  |  | TAL1 |  |
| L1CAM |  |  |  | L1CAM |  |
| MDH2 |  |  |  | MDH2 |  |
| MMEL1 |  |  |  | MMEL1 |  |
| IFNL3 |  |  |  | IFNL3 |  |
| MAPK10 |  |  |  | MAPK10 |  |
| IFNGR1 |  |  |  | IFNGR1 |  |
| NCOR2 |  |  |  | NCOR2 |  |
| CUX1 |  |  |  | CUX1 |  |
| UCP2 |  |  |  | UCP2 |  |
| UGT1A3 |  |  |  | UGT1A3 |  |
| ETV5 |  |  |  | ETV5 |  |
| SLCO1A2 |  |  |  | SLCO1A2 |  |
| IFI27 |  |  |  | IFI27 |  |
| G6PC3 |  |  |  | G6PC3 |  |
| NDUFA13 |  |  |  | NDUFA13 |  |
| LTF |  |  |  | LTF |  |
| SLC27A5 |  |  |  | SLC27A5 |  |
| PROX1 |  |  |  | PROX1 |  |
| AHSG |  |  |  | AHSG |  |
| HBA2 |  |  |  | HBA2 |  |
| IFNAR2 |  |  |  | IFNAR2 |  |
| EZR |  |  |  | EZR |  |
| MAP3K5 |  |  |  | MAP3K5 |  |
| CEACAM6 |  |  |  | CEACAM6 |  |
| COX5A |  |  |  | COX5A |  |
| MT-CO1 |  |  |  | MT-CO1 |  |
| DLAT |  |  |  | DLAT |  |
| VIPAS39 |  |  |  | VIPAS39 |  |
| CHUK |  |  |  | CHUK |  |
| CADM1 |  |  |  | CADM1 |  |
| FANCB |  |  |  | FANCB |  |
| HES1 |  |  |  | HES1 |  |
| LEF1 |  |  |  | LEF1 |  |
| APEX1 |  |  |  | APEX1 |  |
| SMAD5-AS1 |  |  |  | SMAD5-AS1 |  |
| TREX1 |  |  |  | TREX1 |  |
| NR5A1 |  |  |  | NR5A1 |  |
| PNP |  |  |  | PNP |  |
| ACVR2B |  |  |  | ACVR2B |  |
| TWNK |  |  |  | TWNK |  |
| IL2RG |  |  |  | IL2RG |  |
| PDK1 |  |  |  | PDK1 |  |
| AOX1 |  |  |  | AOX1 |  |
| HBA1 |  |  |  | HBA1 |  |
| TNFRSF13B |  |  |  | TNFRSF13B |  |
| MIR184 |  |  |  | MIR184 |  |
| GRN |  |  |  | GRN |  |
| NR1I3 |  |  |  | NR1I3 |  |
| PRKCB |  |  |  | PRKCB |  |
| MPI |  |  |  | MPI |  |
| CYP2C8 |  |  |  | CYP2C8 |  |
| ACOX2 |  |  |  | ACOX2 |  |
| ACTN4 |  |  |  | ACTN4 |  |
| PEX12 |  |  |  | PEX12 |  |
| MIR486-1 |  |  |  | MIR486-1 |  |
| ARMC5 |  |  |  | ARMC5 |  |
| NUP98 |  |  |  | NUP98 |  |
| ADAMTS13 |  |  |  | ADAMTS13 |  |
| PAX6 |  |  |  | PAX6 |  |
| CTTN |  |  |  | CTTN |  |
| TNPO3 |  |  |  | TNPO3 |  |
| DUSP1 |  |  |  | DUSP1 |  |
| PSMB8 |  |  |  | PSMB8 |  |
| LECT2 |  |  |  | LECT2 |  |
| RB1CC1 |  |  |  | RB1CC1 |  |
| PTH1R |  |  |  | PTH1R |  |
| CETP |  |  |  | CETP |  |
| HSPG2 |  |  |  | HSPG2 |  |
| RGN |  |  |  | RGN |  |
| VCP |  |  |  | VCP |  |
| TRIM32 |  |  |  | TRIM32 |  |
| RRAS2 |  |  |  | RRAS2 |  |
| UGT2B7 |  |  |  | UGT2B7 |  |
| MDM4 |  |  |  | MDM4 |  |
| BID |  |  |  | BID |  |
| CIITA |  |  |  | CIITA |  |
| GALK1 |  |  |  | GALK1 |  |
| ARAF |  |  |  | ARAF |  |
| HK2 |  |  |  | HK2 |  |
| MIR532 |  |  |  | MIR532 |  |
| RANBP2 |  |  |  | RANBP2 |  |
| HNRNPK |  |  |  | HNRNPK |  |
| IDO1 |  |  |  | IDO1 |  |
| BGLAP |  |  |  | BGLAP |  |
| BMPR2 |  |  |  | BMPR2 |  |
| ETFDH |  |  |  | ETFDH |  |
| CCL20 |  |  |  | CCL20 |  |
| LIMA1 |  |  |  | LIMA1 |  |
| MIR99B |  |  |  | MIR99B |  |
| SCARB2 |  |  |  | SCARB2 |  |
| FENDRR |  |  |  | FENDRR |  |
| SMC1A |  |  |  | SMC1A |  |
| COL2A1 |  |  |  | COL2A1 |  |
| INSL6 |  |  |  | INSL6 |  |
| OCLN |  |  |  | OCLN |  |
| SELP |  |  |  | SELP |  |
| PYGM |  |  |  | PYGM |  |
| MIR92A2 |  |  |  | MIR92A2 |  |
| ABCD1 |  |  |  | ABCD1 |  |
| PICALM |  |  |  | PICALM |  |
| AHI1 |  |  |  | AHI1 |  |
| ARSB |  |  |  | ARSB |  |
| LMO1 |  |  |  | LMO1 |  |
| VEGFD |  |  |  | VEGFD |  |
| RYR1 |  |  |  | RYR1 |  |
| CYP11B1 |  |  |  | CYP11B1 |  |
| NES |  |  |  | NES |  |
| TCTN2 |  |  |  | TCTN2 |  |
| ASAH1 |  |  |  | ASAH1 |  |
| HDAC6 |  |  |  | HDAC6 |  |
| CBLB |  |  |  | CBLB |  |
| TNNI3 |  |  |  | TNNI3 |  |
| TMEM237 |  |  |  | TMEM237 |  |
| NOP10 |  |  |  | NOP10 |  |
| GHRH |  |  |  | GHRH |  |
| TH |  |  |  | TH |  |
| PLAG1 |  |  |  | PLAG1 |  |
| SCD |  |  |  | SCD |  |
| BCORL1 |  |  |  | BCORL1 |  |
| SPRED1 |  |  |  | SPRED1 |  |
| NGFR |  |  |  | NGFR |  |
| ACP5 |  |  |  | ACP5 |  |
| MAT2A |  |  |  | MAT2A |  |
| POLGARF |  |  |  | POLGARF |  |
| GPT2 |  |  |  | GPT2 |  |
| TSG101 |  |  |  | TSG101 |  |
| DNM2 |  |  |  | DNM2 |  |
| PBX1 |  |  |  | PBX1 |  |
| FSCN1 |  |  |  | FSCN1 |  |
| MIR425 |  |  |  | MIR425 |  |
| SLCO2B1 |  |  |  | SLCO2B1 |  |
| UNC13D |  |  |  | UNC13D |  |
| KIAA0586 |  |  |  | KIAA0586 |  |
| GNRH1 |  |  |  | GNRH1 |  |
| MIR28 |  |  |  | MIR28 |  |
| MLXIPL |  |  |  | MLXIPL |  |
| HHEX |  |  |  | HHEX |  |
| FOXH1 |  |  |  | FOXH1 |  |
| F9 |  |  |  | F9 |  |
| BICC1 |  |  |  | BICC1 |  |
| CD80 |  |  |  | CD80 |  |
| ACVR1 |  |  |  | ACVR1 |  |
| MIR370 |  |  |  | MIR370 |  |
| FUCA1 |  |  |  | FUCA1 |  |
| TGFB3 |  |  |  | TGFB3 |  |
| TNFSF15 |  |  |  | TNFSF15 |  |
| HOGA1 |  |  |  | HOGA1 |  |
| SULT1A1 |  |  |  | SULT1A1 |  |
| PGM3 |  |  |  | PGM3 |  |
| SI |  |  |  | SI |  |
| SHMT1 |  |  |  | SHMT1 |  |
| TNFRSF11B |  |  |  | TNFRSF11B |  |
| MUC4 |  |  |  | MUC4 |  |
| BMI1 |  |  |  | BMI1 |  |
| BTRC |  |  |  | BTRC |  |
| LOC130001603 |  |  |  | LOC130001603 |  |
| PFKM |  |  |  | PFKM |  |
| ADK |  |  |  | ADK |  |
| CYBA |  |  |  | CYBA |  |
| ROBO1 |  |  |  | ROBO1 |  |
| MTAP |  |  |  | MTAP |  |
| SKIL |  |  |  | SKIL |  |
| FGF10 |  |  |  | FGF10 |  |
| MYLK |  |  |  | MYLK |  |
| AREG |  |  |  | AREG |  |
| DIABLO |  |  |  | DIABLO |  |
| ACACA |  |  |  | ACACA |  |
| DPM1 |  |  |  | DPM1 |  |
| ANPEP |  |  |  | ANPEP |  |
| COL4A3 |  |  |  | COL4A3 |  |
| PLK1 |  |  |  | PLK1 |  |
| MIR663A |  |  |  | MIR663A |  |
| MKKS |  |  |  | MKKS |  |
| CYB5A |  |  |  | CYB5A |  |
| SPARC |  |  |  | SPARC |  |
| F13A1 |  |  |  | F13A1 |  |
| ABCC6 |  |  |  | ABCC6 |  |
| TJP1 |  |  |  | TJP1 |  |
| TCTN1 |  |  |  | TCTN1 |  |
| PTP4A3 |  |  |  | PTP4A3 |  |
| CD99 |  |  |  | CD99 |  |
| STAR |  |  |  | STAR |  |
| TNFRSF11A |  |  |  | TNFRSF11A |  |
| ING1 |  |  |  | ING1 |  |
| TINCR |  |  |  | TINCR |  |
| MIR1225 |  |  |  | MIR1225 |  |
| RAB27A |  |  |  | RAB27A |  |
| RASGRP1 |  |  |  | RASGRP1 |  |
| CCL3 |  |  |  | CCL3 |  |
| MMP13 |  |  |  | MMP13 |  |
| CR2 |  |  |  | CR2 |  |
| SFRP1 |  |  |  | SFRP1 |  |
| SLC22A1 |  |  |  | SLC22A1 |  |
| IFT140 |  |  |  | IFT140 |  |
| CDC25C |  |  |  | CDC25C |  |
| NR0B1 |  |  |  | NR0B1 |  |
| CUBN |  |  |  | CUBN |  |
| PEG10 |  |  |  | PEG10 |  |
| AICDA |  |  |  | AICDA |  |
| BAK1 |  |  |  | BAK1 |  |
| RBBP8 |  |  |  | RBBP8 |  |
| PLIN1 |  |  |  | PLIN1 |  |
| SIX1 |  |  |  | SIX1 |  |
| POSTN |  |  |  | POSTN |  |
| LINC01133 |  |  |  | LINC01133 |  |
| TRIM24 |  |  |  | TRIM24 |  |
| DDB2 |  |  |  | DDB2 |  |
| EIF4E |  |  |  | EIF4E |  |
| EIF2AK2 |  |  |  | EIF2AK2 |  |
| PARN |  |  |  | PARN |  |
| GZMB |  |  |  | GZMB |  |
| UICLM |  |  |  | UICLM |  |
| EPHA2 |  |  |  | EPHA2 |  |
| RELN |  |  |  | RELN |  |
| CFI |  |  |  | CFI |  |
| VIP |  |  |  | VIP |  |
| ZFHX3 |  |  |  | ZFHX3 |  |
| IL15 |  |  |  | IL15 |  |
| CKB |  |  |  | CKB |  |
| SLC25A15 |  |  |  | SLC25A15 |  |
| TRAP1 |  |  |  | TRAP1 |  |
| MIR196A1 |  |  |  | MIR196A1 |  |
| C3 |  |  |  | C3 |  |
| SAA1 |  |  |  | SAA1 |  |
| SFTA3 |  |  |  | SFTA3 |  |
| RPGRIP1 |  |  |  | RPGRIP1 |  |
| MIR29B1 |  |  |  | MIR29B1 |  |
| NUP214 |  |  |  | NUP214 |  |
| FOSL1 |  |  |  | FOSL1 |  |
| ASTILCS |  |  |  | ASTILCS |  |
| PAH |  |  |  | PAH |  |
| PRSS1 |  |  |  | PRSS1 |  |
| MIR346 |  |  |  | MIR346 |  |
| ACADS |  |  |  | ACADS |  |
| STS |  |  |  | STS |  |
| GLIS3 |  |  |  | GLIS3 |  |
| GNPTAB |  |  |  | GNPTAB |  |
| RRM2B |  |  |  | RRM2B |  |
| TGIF1 |  |  |  | TGIF1 |  |
| CAVIN1 |  |  |  | CAVIN1 |  |
| PEX3 |  |  |  | PEX3 |  |
| GCKR |  |  |  | GCKR |  |
| MIR339 |  |  |  | MIR339 |  |
| CEACAM1 |  |  |  | CEACAM1 |  |
| HDAC2 |  |  |  | HDAC2 |  |
| RUNX1T1 |  |  |  | RUNX1T1 |  |
| KLF11 |  |  |  | KLF11 |  |
| FOXE1 |  |  |  | FOXE1 |  |
| FGF21 |  |  |  | FGF21 |  |
| CTNNA3 |  |  |  | CTNNA3 |  |
| RNY1 |  |  |  | RNY1 |  |
| APOL1 |  |  |  | APOL1 |  |
| ETV4 |  |  |  | ETV4 |  |
| CD86 |  |  |  | CD86 |  |
| THY1 |  |  |  | THY1 |  |
| MFN2 |  |  |  | MFN2 |  |
| FOXP2 |  |  |  | FOXP2 |  |
| LBR |  |  |  | LBR |  |
| LOC126860438 |  |  |  | LOC126860438 |  |
| INHA |  |  |  | INHA |  |
| CD276 |  |  |  | CD276 |  |
| DLK1 |  |  |  | DLK1 |  |
| SCT |  |  |  | SCT |  |
| ARID2 |  |  |  | ARID2 |  |
| MIR675 |  |  |  | MIR675 |  |
| HK1 |  |  |  | HK1 |  |
| TBX20 |  |  |  | TBX20 |  |
| CHD1L |  |  |  | CHD1L |  |
| GLI3 |  |  |  | GLI3 |  |
| MIR495 |  |  |  | MIR495 |  |
| ZNF423 |  |  |  | ZNF423 |  |
| LIPE |  |  |  | LIPE |  |
| SLC51A |  |  |  | SLC51A |  |
| CACNA2D1 |  |  |  | CACNA2D1 |  |
| DAPK1 |  |  |  | DAPK1 |  |
| SEMA4D |  |  |  | SEMA4D |  |
| SERPINA6 |  |  |  | SERPINA6 |  |
| SPINT2 |  |  |  | SPINT2 |  |
| SUMF1 |  |  |  | SUMF1 |  |
| AIFM1 |  |  |  | AIFM1 |  |
| EDN3 |  |  |  | EDN3 |  |
| CD163 |  |  |  | CD163 |  |
| HGSNAT |  |  |  | HGSNAT |  |
| KLF1 |  |  |  | KLF1 |  |
| SMC3 |  |  |  | SMC3 |  |
| F10 |  |  |  | F10 |  |
| LIN28B |  |  |  | LIN28B |  |
| NGF |  |  |  | NGF |  |
| SERPINB3 |  |  |  | SERPINB3 |  |
| CEP83 |  |  |  | CEP83 |  |
| CD55 |  |  |  | CD55 |  |
| RIT1 |  |  |  | RIT1 |  |
| HMGA1 |  |  |  | HMGA1 |  |
| LGALS1 |  |  |  | LGALS1 |  |
| MIR124-3 |  |  |  | MIR124-3 |  |
| ITK |  |  |  | ITK |  |
| MECP2 |  |  |  | MECP2 |  |
| BLNK |  |  |  | BLNK |  |
| TALDO1 |  |  |  | TALDO1 |  |
| FAM13A |  |  |  | FAM13A |  |
| FOXP1 |  |  |  | FOXP1 |  |
| SALL4 |  |  |  | SALL4 |  |
| NODAL |  |  |  | NODAL |  |
| SPTBN1 |  |  |  | SPTBN1 |  |
| HAND1 |  |  |  | HAND1 |  |
| CTNND1 |  |  |  | CTNND1 |  |
| TRPV1 |  |  |  | TRPV1 |  |
| CYP27B1 |  |  |  | CYP27B1 |  |
| EPHA3 |  |  |  | EPHA3 |  |
| HLA-G |  |  |  | HLA-G |  |
| OXA1L |  |  |  | OXA1L |  |
| PRKACB |  |  |  | PRKACB |  |
| GCGR |  |  |  | GCGR |  |
| LTBP4 |  |  |  | LTBP4 |  |
| LGR5 |  |  |  | LGR5 |  |
| ITGB3 |  |  |  | ITGB3 |  |
| CHD1 |  |  |  | CHD1 |  |
| MIR134 |  |  |  | MIR134 |  |
| ALDH9A1 |  |  |  | ALDH9A1 |  |
| PTHLH |  |  |  | PTHLH |  |
| CLEC4M |  |  |  | CLEC4M |  |
| DCK |  |  |  | DCK |  |
| CTH |  |  |  | CTH |  |
| MIR491 |  |  |  | MIR491 |  |
| KDM5C |  |  |  | KDM5C |  |
| MAOA |  |  |  | MAOA |  |
| DSG2 |  |  |  | DSG2 |  |
| NAT1 |  |  |  | NAT1 |  |
| WEE1 |  |  |  | WEE1 |  |
| SULT2A1 |  |  |  | SULT2A1 |  |
| MSLN |  |  |  | MSLN |  |
| ACD |  |  |  | ACD |  |
| ETFA |  |  |  | ETFA |  |
| LYST |  |  |  | LYST |  |
| LOC107303338 |  |  |  | LOC107303338 |  |
| NBAT1 |  |  |  | NBAT1 |  |
| IL22 |  |  |  | IL22 |  |
| DLEC1 |  |  |  | DLEC1 |  |
| ATP6AP2 |  |  |  | ATP6AP2 |  |
| RPL11 |  |  |  | RPL11 |  |
| INHBA |  |  |  | INHBA |  |
| OSM |  |  |  | OSM |  |
| TMPRSS6 |  |  |  | TMPRSS6 |  |
| CTSK |  |  |  | CTSK |  |
| PIGA |  |  |  | PIGA |  |
| CYP11A1 |  |  |  | CYP11A1 |  |
| CTAG1B |  |  |  | CTAG1B |  |
| MYO18A |  |  |  | MYO18A |  |
| CRLF2 |  |  |  | CRLF2 |  |
| ACE2 |  |  |  | ACE2 |  |
| CD5 |  |  |  | CD5 |  |
| SNORD15A |  |  |  | SNORD15A |  |
| MT-TT |  |  |  | MT-TT |  |
| NFATC1 |  |  |  | NFATC1 |  |
| YBX1 |  |  |  | YBX1 |  |
| RHBDF2 |  |  |  | RHBDF2 |  |
| TMEM107 |  |  |  | TMEM107 |  |
| LMO2 |  |  |  | LMO2 |  |
| ACAD9 |  |  |  | ACAD9 |  |
| DLEU1 |  |  |  | DLEU1 |  |
| CXCR3 |  |  |  | CXCR3 |  |
| TMEM138 |  |  |  | TMEM138 |  |
| APP |  |  |  | APP |  |
| CBLC |  |  |  | CBLC |  |
| PGR-AS1 |  |  |  | PGR-AS1 |  |
| EIF1AX |  |  |  | EIF1AX |  |
| SLC16A1 |  |  |  | SLC16A1 |  |
| ITGAM |  |  |  | ITGAM |  |
| SLC12A1 |  |  |  | SLC12A1 |  |
| EGOT |  |  |  | EGOT |  |
| CHI3L1 |  |  |  | CHI3L1 |  |
| PODXL |  |  |  | PODXL |  |
| RPL5 |  |  |  | RPL5 |  |
| MAGT1 |  |  |  | MAGT1 |  |
| CLCNKB |  |  |  | CLCNKB |  |
| NPHS2 |  |  |  | NPHS2 |  |
| SLPI |  |  |  | SLPI |  |
| BTNL2 |  |  |  | BTNL2 |  |
| ZAP70 |  |  |  | ZAP70 |  |
| CAV3 |  |  |  | CAV3 |  |
| IL3RA |  |  |  | IL3RA |  |
| FURIN |  |  |  | FURIN |  |
| COL4A4 |  |  |  | COL4A4 |  |
| PSEN1 |  |  |  | PSEN1 |  |
| LINC00958 |  |  |  | LINC00958 |  |
| STAT4 |  |  |  | STAT4 |  |
| CDH23 |  |  |  | CDH23 |  |
| TNFSF12 |  |  |  | TNFSF12 |  |
| LRPPRC |  |  |  | LRPPRC |  |
| MTRR |  |  |  | MTRR |  |
| MERTK |  |  |  | MERTK |  |
| GPR35 |  |  |  | GPR35 |  |
| EHHADH |  |  |  | EHHADH |  |
| NOTCH4 |  |  |  | NOTCH4 |  |
| MAPT |  |  |  | MAPT |  |
| CD38 |  |  |  | CD38 |  |
| CES2 |  |  |  | CES2 |  |
| PXDN |  |  |  | PXDN |  |
| B9D2 |  |  |  | B9D2 |  |
| NAGS |  |  |  | NAGS |  |
| LOC107303340 |  |  |  | LOC107303340 |  |
| GC |  |  |  | GC |  |
| NUTM1 |  |  |  | NUTM1 |  |
| GSTM3 |  |  |  | GSTM3 |  |
| TNFRSF6B |  |  |  | TNFRSF6B |  |
| PTGS1 |  |  |  | PTGS1 |  |
| LINC01234 |  |  |  | LINC01234 |  |
| STIM1 |  |  |  | STIM1 |  |
| GLS |  |  |  | GLS |  |
| MCM4 |  |  |  | MCM4 |  |
| HSPA8 |  |  |  | HSPA8 |  |
| MIR4435-2HG |  |  |  | MIR4435-2HG |  |
| ITGA6 |  |  |  | ITGA6 |  |
| TFF1 |  |  |  | TFF1 |  |
| CSPP1 |  |  |  | CSPP1 |  |
| U2AF2 |  |  |  | U2AF2 |  |
| GLI2 |  |  |  | GLI2 |  |
| ABCD3 |  |  |  | ABCD3 |  |
| NQO2 |  |  |  | NQO2 |  |
| MED1 |  |  |  | MED1 |  |
| HOXA9 |  |  |  | HOXA9 |  |
| TGFBR3 |  |  |  | TGFBR3 |  |
| CHD4 |  |  |  | CHD4 |  |
| MIR455 |  |  |  | MIR455 |  |
| FAP |  |  |  | FAP |  |
| MLLT10 |  |  |  | MLLT10 |  |
| SLC11A1 |  |  |  | SLC11A1 |  |
| NR0B2 |  |  |  | NR0B2 |  |
| LRAT |  |  |  | LRAT |  |
| ALCAM |  |  |  | ALCAM |  |
| PHF6 |  |  |  | PHF6 |  |
| NPHS1 |  |  |  | NPHS1 |  |
| ITGAV |  |  |  | ITGAV |  |
| CRKL |  |  |  | CRKL |  |
| APPL1 |  |  |  | APPL1 |  |
| LINC00511 |  |  |  | LINC00511 |  |
| BCL3 |  |  |  | BCL3 |  |
| AK2 |  |  |  | AK2 |  |
| ITGA3 |  |  |  | ITGA3 |  |
| BCL11B |  |  |  | BCL11B |  |
| HMGCS2 |  |  |  | HMGCS2 |  |
| RREB1 |  |  |  | RREB1 |  |
| SLC12A3 |  |  |  | SLC12A3 |  |
| HSD11B1 |  |  |  | HSD11B1 |  |
| LUCAT1 |  |  |  | LUCAT1 |  |
| CDC42 |  |  |  | CDC42 |  |
| SAMD9L |  |  |  | SAMD9L |  |
| LRRK2 |  |  |  | LRRK2 |  |
| CTSA |  |  |  | CTSA |  |
| AGK |  |  |  | AGK |  |
| SUCLG1 |  |  |  | SUCLG1 |  |
| CEP41 |  |  |  | CEP41 |  |
| IKZF3 |  |  |  | IKZF3 |  |
| MMP11 |  |  |  | MMP11 |  |
| KRIT1 |  |  |  | KRIT1 |  |
| MIR511 |  |  |  | MIR511 |  |
| ATP6AP1 |  |  |  | ATP6AP1 |  |
| TAFAZZIN |  |  |  | TAFAZZIN |  |
| CCR1 |  |  |  | CCR1 |  |
| PAX4 |  |  |  | PAX4 |  |
| PRLR |  |  |  | PRLR |  |
| SIX5 |  |  |  | SIX5 |  |
| SERPINB5 |  |  |  | SERPINB5 |  |
| TTPA |  |  |  | TTPA |  |
| MIR340 |  |  |  | MIR340 |  |
| MAPK9 |  |  |  | MAPK9 |  |
| FUT8 |  |  |  | FUT8 |  |
| FGF1 |  |  |  | FGF1 |  |
| XRCC6 |  |  |  | XRCC6 |  |
| NEK2 |  |  |  | NEK2 |  |
| NAA10 |  |  |  | NAA10 |  |
| LINC01772 |  |  |  | LINC01772 |  |
| NAB2 |  |  |  | NAB2 |  |
| C4A |  |  |  | C4A |  |
| SAMD9 |  |  |  | SAMD9 |  |
| MAF |  |  |  | MAF |  |
| PHGDH |  |  |  | PHGDH |  |
| IGF2BP2 |  |  |  | IGF2BP2 |  |
| PTP4A1 |  |  |  | PTP4A1 |  |
| GNS |  |  |  | GNS |  |
| DEK |  |  |  | DEK |  |
| CASZ1 |  |  |  | CASZ1 |  |
| PLCG2 |  |  |  | PLCG2 |  |
| MIR381 |  |  |  | MIR381 |  |
| TRB |  |  |  | TRB |  |
| MAN2B1 |  |  |  | MAN2B1 |  |
| MAP3K14 |  |  |  | MAP3K14 |  |
| PGK1 |  |  |  | PGK1 |  |
| EPB41L4A-DT |  |  |  | EPB41L4A-DT |  |
| TRAF7 |  |  |  | TRAF7 |  |
| BTD |  |  |  | BTD |  |
| TUBB |  |  |  | TUBB |  |
| CLCN5 |  |  |  | CLCN5 |  |
| F7 |  |  |  | F7 |  |
| GSTA2 |  |  |  | GSTA2 |  |
| MIR574 |  |  |  | MIR574 |  |
| ONECUT2 |  |  |  | ONECUT2 |  |
| PRKCZ |  |  |  | PRKCZ |  |
| SLC10A2 |  |  |  | SLC10A2 |  |
| CYP3A7 |  |  |  | CYP3A7 |  |
| LRP1 |  |  |  | LRP1 |  |
| UTP4 |  |  |  | UTP4 |  |
| CIDEC |  |  |  | CIDEC |  |
| MIR502 |  |  |  | MIR502 |  |
| PEMT |  |  |  | PEMT |  |
| WWTR1 |  |  |  | WWTR1 |  |
| KCNJ1 |  |  |  | KCNJ1 |  |
| HOXA10 |  |  |  | HOXA10 |  |
| IL1R1 |  |  |  | IL1R1 |  |
| STK4 |  |  |  | STK4 |  |
| STAT2 |  |  |  | STAT2 |  |
| NAMPT |  |  |  | NAMPT |  |
| CXCL1 |  |  |  | CXCL1 |  |
| GPD1 |  |  |  | GPD1 |  |
| CREB3L3 |  |  |  | CREB3L3 |  |
| KIR3DL1 |  |  |  | KIR3DL1 |  |
| RNF220 |  |  |  | RNF220 |  |
| SLC35C1 |  |  |  | SLC35C1 |  |
| DUXAP9 |  |  |  | DUXAP9 |  |
| PINX1 |  |  |  | PINX1 |  |
| PITX2 |  |  |  | PITX2 |  |
| MVP |  |  |  | MVP |  |
| NR2F2 |  |  |  | NR2F2 |  |
| PDE11A |  |  |  | PDE11A |  |
| SCO2 |  |  |  | SCO2 |  |
| APOA5 |  |  |  | APOA5 |  |
| CNTNAP2 |  |  |  | CNTNAP2 |  |
| HAX1 |  |  |  | HAX1 |  |
| CYP21A2 |  |  |  | CYP21A2 |  |
| KLRK1 |  |  |  | KLRK1 |  |
| TRIM28 |  |  |  | TRIM28 |  |
| GCDH |  |  |  | GCDH |  |
| CARS2 |  |  |  | CARS2 |  |
| FGF7 |  |  |  | FGF7 |  |
| DLD |  |  |  | DLD |  |
| GSTA1 |  |  |  | GSTA1 |  |
| AP3B1 |  |  |  | AP3B1 |  |
| ZRSR2 |  |  |  | ZRSR2 |  |
| AKR1B10 |  |  |  | AKR1B10 |  |
| SCO1 |  |  |  | SCO1 |  |
| MOGS |  |  |  | MOGS |  |
| E2F4 |  |  |  | E2F4 |  |
| ADM |  |  |  | ADM |  |
| CAD |  |  |  | CAD |  |
| FBXO11 |  |  |  | FBXO11 |  |
| MIR33B |  |  |  | MIR33B |  |
| NCOA3 |  |  |  | NCOA3 |  |
| MBD4 |  |  |  | MBD4 |  |
| PTPN12 |  |  |  | PTPN12 |  |
| SPI1 |  |  |  | SPI1 |  |
| TK2 |  |  |  | TK2 |  |
| COQ6 |  |  |  | COQ6 |  |
| SOX17 |  |  |  | SOX17 |  |
| ACAT1 |  |  |  | ACAT1 |  |
| TAF15 |  |  |  | TAF15 |  |
| TNFRSF9 |  |  |  | TNFRSF9 |  |
| ASGR2 |  |  |  | ASGR2 |  |
| NKX2-5 |  |  |  | NKX2-5 |  |
| RPS19 |  |  |  | RPS19 |  |
| PCCA |  |  |  | PCCA |  |
| PCA3 |  |  |  | PCA3 |  |
| HLA-C |  |  |  | HLA-C |  |
| GCG |  |  |  | GCG |  |
| HSD3B2 |  |  |  | HSD3B2 |  |
| PRKG1 |  |  |  | PRKG1 |  |
| BLVRA |  |  |  | BLVRA |  |
| SLC51B |  |  |  | SLC51B |  |
| PTPRJ |  |  |  | PTPRJ |  |
| PSMB9 |  |  |  | PSMB9 |  |
| SLC7A7 |  |  |  | SLC7A7 |  |
| MAGEA1 |  |  |  | MAGEA1 |  |
| PRDX5 |  |  |  | PRDX5 |  |
| PAX3 |  |  |  | PAX3 |  |
| ANKRD26 |  |  |  | ANKRD26 |  |
| CASC8 |  |  |  | CASC8 |  |
| LATS1 |  |  |  | LATS1 |  |
| BLK |  |  |  | BLK |  |
| XRCC4 |  |  |  | XRCC4 |  |
| PCBD1 |  |  |  | PCBD1 |  |
| UQCRB |  |  |  | UQCRB |  |
| MDK |  |  |  | MDK |  |
| GRP |  |  |  | GRP |  |
| PTPN1 |  |  |  | PTPN1 |  |
| USH2A |  |  |  | USH2A |  |
| BGN |  |  |  | BGN |  |
| HDAC3 |  |  |  | HDAC3 |  |
| TTC8 |  |  |  | TTC8 |  |
| ALDH7A1 |  |  |  | ALDH7A1 |  |
| ETFB |  |  |  | ETFB |  |
| CCK |  |  |  | CCK |  |
| ALG5 |  |  |  | ALG5 |  |
| MIR302C |  |  |  | MIR302C |  |
| LYZ |  |  |  | LYZ |  |
| IGKC |  |  |  | IGKC |  |
| AKR1C4 |  |  |  | AKR1C4 |  |
| AQP1 |  |  |  | AQP1 |  |
| RIPK1 |  |  |  | RIPK1 |  |
| KLF2 |  |  |  | KLF2 |  |
| PRDX1 |  |  |  | PRDX1 |  |
| USP53 |  |  |  | USP53 |  |
| CCDC115 |  |  |  | CCDC115 |  |
| GSR |  |  |  | GSR |  |
| KDM1A |  |  |  | KDM1A |  |
| PPP1CB |  |  |  | PPP1CB |  |
| MNX1-AS1 |  |  |  | MNX1-AS1 |  |
| MIR135B |  |  |  | MIR135B |  |
| GAMT |  |  |  | GAMT |  |
| COQ2 |  |  |  | COQ2 |  |
| SLC26A4 |  |  |  | SLC26A4 |  |
| CASC9 |  |  |  | CASC9 |  |
| SULT1E1 |  |  |  | SULT1E1 |  |
| SPOP |  |  |  | SPOP |  |
| GFM1 |  |  |  | GFM1 |  |
| LPIN2 |  |  |  | LPIN2 |  |
| PLIN2 |  |  |  | PLIN2 |  |
| MYL2 |  |  |  | MYL2 |  |
| FGF20 |  |  |  | FGF20 |  |
| PHB2 |  |  |  | PHB2 |  |
| AMBP |  |  |  | AMBP |  |
| SMARCAL1 |  |  |  | SMARCAL1 |  |
| LPIN1 |  |  |  | LPIN1 |  |
| ICOS |  |  |  | ICOS |  |
| ETV1 |  |  |  | ETV1 |  |
| LMBRD1 |  |  |  | LMBRD1 |  |
| MIR129-1 |  |  |  | MIR129-1 |  |
| UPK3A |  |  |  | UPK3A |  |
| NR3C2 |  |  |  | NR3C2 |  |
| LSR |  |  |  | LSR |  |
| HPX |  |  |  | HPX |  |
| EXOC2 |  |  |  | EXOC2 |  |
| DRD2 |  |  |  | DRD2 |  |
| MT-TL1 |  |  |  | MT-TL1 |  |
| TNNT2 |  |  |  | TNNT2 |  |
| ANGPTL3 |  |  |  | ANGPTL3 |  |
| APTX |  |  |  | APTX |  |
| TRAF5 |  |  |  | TRAF5 |  |
| MSH5 |  |  |  | MSH5 |  |
| NPY |  |  |  | NPY |  |
| SLC4A1 |  |  |  | SLC4A1 |  |
| SPG7 |  |  |  | SPG7 |  |
| SREBF2 |  |  |  | SREBF2 |  |
| CHGB |  |  |  | CHGB |  |
| HYAL1 |  |  |  | HYAL1 |  |
| RHOC |  |  |  | RHOC |  |
| PRKAA2 |  |  |  | PRKAA2 |  |
| SNCA |  |  |  | SNCA |  |
|  |  |  |  | FMO1 |  |
|  |  |  |  | FMO4 |  |
|  |  |  |  | LEAP2 |  |
|  |  |  |  | COX7A2 |  |
|  |  |  |  | NAFLD2 |  |
|  |  |  |  | RNASE2 |  |
|  |  |  |  | NAFLD1 |  |
|  |  |  |  | SLC25A6 |  |
|  |  |  |  | JAK-2 |  |
|  |  |  |  | S1PR1 |  |
|  |  |  |  | CTLA-4 |  |
|  |  |  |  | HB |  |
|  |  |  |  | PD-L1 |  |
|  |  |  |  | PD-1 |  |
|  |  |  |  | TLR7 |  |
|  |  |  |  | TOP2 |  |
|  |  |  |  | hDNA |  |
|  |  |  |  | ADORA3 |  |
|  |  |  |  | CSNK2A1 |  |
|  |  |  |  | FLT-4 |  |
|  |  |  |  | TRAIL-R2 |  |
|  |  |  |  | KIF11 |  |
|  |  |  |  | MKNK2 |  |
|  |  |  |  | MKNK1 |  |
|  |  |  |  | SPHK2 |  |
|  |  |  |  | EGFR vIII |  |
|  |  |  |  | MYCBP |  |
|  |  |  |  | FACT |  |
|  |  |  |  | RORG |  |
|  |  |  |  | CRBN |  |
|  |  |  |  | MIR34 |  |
|  |  |  |  | CD80/PD-L1 PPI |  |
|  |  |  |  | PD-1/PD-L1 PPI |  |
|  |  |  |  | RORA |  |
|  |  |  |  | KMT5A |  |
|  |  |  |  | DDEFL1 |  |
|  |  |  |  | IL27 |  |
|  |  |  |  | IFNA |  |
|  |  |  |  | RAR |  |
|  |  |  |  | CEBPA? |  |
|  |  |  |  | FGFR |  |
|  |  |  |  | HLA-A02/AFP |  |
|  |  |  |  | PK |  |
|  |  |  |  | S1PR |  |
|  |  |  |  | CSK |  |
|  |  |  |  | CSNK2 |  |
|  |  |  |  | STK |  |
|  |  |  |  | TCR |  |
|  |  |  |  | Wnt pathway |  |
|  |  |  |  | CT47A12 |  |
|  |  |  |  | CT45A8 |  |
|  |  |  |  | CT45A9 |  |
|  |  |  |  | CT45A10 |  |
|  |  |  |  | CT45A7 |  |
|  |  |  |  | CT47A1 |  |
|  |  |  |  | CT47A11 |  |
|  |  |  |  | CT47A4 |  |
|  |  |  |  | CT47A6 |  |
|  |  |  |  | CT47B1 |  |
|  |  |  |  | CT47A10 |  |
|  |  |  |  | CT47A3 |  |
|  |  |  |  | CT47A5 |  |
|  |  |  |  | CT47A7 |  |
|  |  |  |  | CT47A8 |  |
|  |  |  |  | CT47A9 |  |
|  |  |  |  | CT45A2 |  |
|  |  |  |  | CT45A6 |  |
|  |  |  |  | CT45A5 |  |
|  |  |  |  | COLCA2 |  |
|  |  |  |  | PRAC1 |  |
|  |  |  |  | CAGE1 |  |
|  |  |  |  | CT45B1P |  |
|  |  |  |  | CT83 |  |
|  |  |  |  | KIAA0100 |  |
|  |  |  |  | LTO1 |  |
|  |  |  |  | PAGE5 |  |
|  |  |  |  | CTAG2 |  |
|  |  |  |  | XAGE3 |  |
|  |  |  |  | NTPCR |  |
|  |  |  |  | LETMD1 |  |
|  |  |  |  | GREB1 |  |
|  |  |  |  | SMIM22 |  |
|  |  |  |  | DERPC |  |
|  |  |  |  | DSCR8 |  |
|  |  |  |  | CTAGE1 |  |
|  |  |  |  | DDX53 |  |
|  |  |  |  | CT62 |  |
|  |  |  |  | VENTXP1 |  |
|  |  |  |  | BCAR3 |  |
|  |  |  |  | ANKRD45 |  |
|  |  |  |  | GAGE4 |  |
|  |  |  |  | GAGE5 |  |
|  |  |  |  | GAGE6 |  |
|  |  |  |  | C20orf85 |  |
|  |  |  |  | C1orf74 |  |
|  |  |  |  | C4orf46 |  |
|  |  |  |  | GAGE7 |  |
|  |  |  |  | HEPN1 |  |
|  |  |  |  | SNCG |  |
|  |  |  |  | ARMC3 |  |
|  |  |  |  | HEATR6 |  |
|  |  |  |  | BAGE |  |
|  |  |  |  | CTAG1A |  |
|  |  |  |  | ANKRD30BP2 |  |
|  |  |  |  | SPANXN3 |  |
|  |  |  |  | SPANXN2 |  |
|  |  |  |  | SPANXN5 |  |
|  |  |  |  | BAGE5 |  |
|  |  |  |  | PBOV1 |  |
|  |  |  |  | SPANXB1 |  |
|  |  |  |  | FAM168A |  |
|  |  |  |  | XAGE5 |  |
|  |  |  |  | MAGEB3 |  |
|  |  |  |  | RMC1 |  |
|  |  |  |  | DPPA2 |  |
|  |  |  |  | XAGE2 |  |
|  |  |  |  | CDK2AP2 |  |
|  |  |  |  | SPAG9 |  |
|  |  |  |  | KNL1 |  |
|  |  |  |  | GKN2 |  |
|  |  |  |  | TCIM |  |
|  |  |  |  | LUZP4 |  |
|  |  |  |  | CT55 |  |
|  |  |  |  | RBM46 |  |
|  |  |  |  | SPANXN4 |  |
|  |  |  |  | SPANXN1 |  |
|  |  |  |  | LYPD6B |  |
|  |  |  |  | MAGEA9 |  |
|  |  |  |  | POTEC |  |
|  |  |  |  | NXF2 |  |
|  |  |  |  | SAGE1 |  |
|  |  |  |  | BAGE4 |  |
|  |  |  |  | GAGE8 |  |
|  |  |  |  | BAGE2 |  |
|  |  |  |  | ACTL8 |  |
|  |  |  |  | BLID |  |
|  |  |  |  | MAGEB4 |  |
|  |  |  |  | SPANXD |  |
|  |  |  |  | CCDC33 |  |
|  |  |  |  | POTEA |  |
|  |  |  |  | GAGE1 |  |
|  |  |  |  | CSAG1 |  |
|  |  |  |  | CCDC110 |  |
|  |  |  |  | HID1 |  |
|  |  |  |  | BAGE3 |  |
|  |  |  |  | CCDC62 |  |
|  |  |  |  | ROPN1 |  |
|  |  |  |  | POTEG |  |
|  |  |  |  | GAGE2A |  |
|  |  |  |  | TULP2 |  |
|  |  |  |  | HSPB9 |  |
|  |  |  |  | PRAC2 |  |
|  |  |  |  | CENPW |  |
|  |  |  |  | FMR1NB |  |
|  |  |  |  | MAGEC2 |  |
|  |  |  |  | ICE2 |  |
|  |  |  |  | TENT5D |  |
|  |  |  |  | POTED |  |
|  |  |  |  | POTEH |  |
|  |  |  |  | VOPPI |  |
|  |  |  |  | MAGEA3 |  |
|  |  |  |  | FATE1 |  |
|  |  |  |  | MAGEC3 |  |
|  |  |  |  | CSAG2 |  |
|  |  |  |  | GOLGA6L2 |  |
|  |  |  |  | HORMAD1 |  |
|  |  |  |  | RTL6 |  |
|  |  |  |  | GPATCH2 |  |
|  |  |  |  | MAGEA10 |  |
|  |  |  |  | CIP2A |  |
|  |  |  |  | SPANXC |  |
|  |  |  |  | SPANXA1 |  |
|  |  |  |  | MAGEA2 |  |
|  |  |  |  | POTEE |  |
|  |  |  |  | PRSS54 |  |
|  |  |  |  | OCIAD2 |  |
|  |  |  |  | ODF3 |  |
|  |  |  |  | LIPI |  |
|  |  |  |  | CPXCR1 |  |
|  |  |  |  | SLC49A4 |  |
|  |  |  |  | ENTR1 |  |
|  |  |  |  | PASD1 |  |
|  |  |  |  | SPINK7 |  |
|  |  |  |  | BRMS1L |  |
|  |  |  |  | LRATD2 |  |
|  |  |  |  | OVCA2 |  |
|  |  |  |  | AGR3 |  |
|  |  |  |  | ODF1 |  |
|  |  |  |  | MAGEB6 |  |
|  |  |  |  | DCAF12 |  |
|  |  |  |  | DEPDC1B |  |
|  |  |  |  | SLCO6A1 |  |
|  |  |  |  | TEX101 |  |
|  |  |  |  | TBC1D3 |  |
|  |  |  |  | PLAC1 |  |
|  |  |  |  | GREB1L |  |
|  |  |  |  | LDOC1 |  |
|  |  |  |  | MAGEC1 |  |
|  |  |  |  | TMEM108 |  |
|  |  |  |  | ZNF165 |  |
|  |  |  |  | SPATA19 |  |
|  |  |  |  | SSX2 |  |
|  |  |  |  | SPA17 |  |
|  |  |  |  | TMEFF1 |  |
|  |  |  |  | C14orf93 |  |
|  |  |  |  | XAGE1A |  |
|  |  |  |  | NLRP4 |  |
|  |  |  |  | ANKRD30A |  |
|  |  |  |  | PNMA3 |  |
|  |  |  |  | MAGEB2 |  |
|  |  |  |  | ACRBP |  |
|  |  |  |  | RHOXF2 |  |
|  |  |  |  | EPPIN |  |
|  |  |  |  | SLC4A1AP |  |
|  |  |  |  | COX6B2 |  |
|  |  |  |  | SPEF2 |  |
|  |  |  |  | ECRG4 |  |
|  |  |  |  | PRSS50 |  |
|  |  |  |  | ODF4 |  |
|  |  |  |  | MORC1 |  |
|  |  |  |  | BRINP1 |  |
|  |  |  |  | TMEFF2 |  |
|  |  |  |  | CALR3 |  |
|  |  |  |  | FAM133A |  |
|  |  |  |  | POTEB |  |
|  |  |  |  | MAGEB5 |  |
|  |  |  |  | HELZ |  |
|  |  |  |  | MAGED2 |  |
|  |  |  |  | THEG |  |
|  |  |  |  | DDX43 |  |
|  |  |  |  | MAGEA12 |  |
|  |  |  |  | CEP55 |  |
|  |  |  |  | SSX1 |  |
|  |  |  |  | SEMG1 |  |
|  |  |  |  | MACC1 |  |
|  |  |  |  | BRMS1 |  |
|  |  |  |  | MAGEA11 |  |
|  |  |  |  | MAGEA6 |  |
|  |  |  |  | TFDP3 |  |
|  |  |  |  | IGSF11 |  |
|  |  |  |  | FTHL17 |  |
|  |  |  |  | MAGEA8 |  |
|  |  |  |  | LEMD1 |  |
|  |  |  |  | HIC1 |  |
|  |  |  |  | PBK |  |
|  |  |  |  | DKKL1 |  |
|  |  |  |  | MRPS11 |  |
|  |  |  |  | IL13RA2 |  |
|  |  |  |  | LY6K |  |
|  |  |  |  | SSX2B |  |
|  |  |  |  | CRISP2 |  |
|  |  |  |  | NOL4 |  |
|  |  |  |  | ELOVL4 |  |
|  |  |  |  | MAGEB1 |  |
|  |  |  |  | SYCE1 |  |
|  |  |  |  | FILIP1L |  |
|  |  |  |  | TEX14 |  |
|  |  |  |  | OTOA |  |
|  |  |  |  | LDHC |  |
|  |  |  |  | BLCAP |  |
|  |  |  |  | TEX15 |  |
|  |  |  |  | SPAG4 |  |
|  |  |  |  | ADAM29 |  |
|  |  |  |  | LSM1 |  |
|  |  |  |  | TSSK6 |  |
|  |  |  |  | OCIAD1 |  |
|  |  |  |  | CCAR2 |  |
|  |  |  |  | GPAT2 |  |
|  |  |  |  | AKAP3 |  |
|  |  |  |  | ATAD2 |  |
|  |  |  |  | OIP5 |  |
|  |  |  |  | TDRD6 |  |
|  |  |  |  | MYCL |  |
|  |  |  |  | PRM1 |  |
|  |  |  |  | PRM2 |  |
|  |  |  |  | TSGA10 |  |
|  |  |  |  | MAGEA4 |  |
|  |  |  |  | SCA1 |  |
|  |  |  |  | ADAM2 |  |
|  |  |  |  | SYCP1 |  |
|  |  |  |  | MCC |  |
|  |  |  |  | NUF2 |  |
|  |  |  |  | TPTE |  |
|  |  |  |  | SPO11 |  |
|  |  |  |  | TDRD1 |  |
|  |  |  |  | TSPY1 |  |
|  |  |  |  | IGF2BP3 |  |
|  |  |  |  | NDUFC2 |  |
|  |  |  |  | TTK |  |
|  |  |  |  | BRDT |  |
|  |  |  |  | KIF20B |  |
|  |  |  |  | SPACA3 |  |
|  |  |  |  | TAF7L |  |
|  |  |  |  | AKAP4 |  |
|  |  |  |  | CASC3 |  |
|  |  |  |  | PRAME |  |
|  |  |  |  | LZTS1 |  |
|  |  |  |  | CABYR |  |
|  |  |  |  | ARX |  |
|  |  |  |  | CTNNA2 |  |
|  |  |  |  | PIWIL2 |  |
|  |  |  |  | KDM5B |  |
|  |  |  |  | CTCFL |  |
|  |  |  |  | MAEL |  |
|  |  |  |  | ODF2 |  |
|  |  |  |  | SYTL2 |  |
|  |  |  |  | BCAR1 |  |


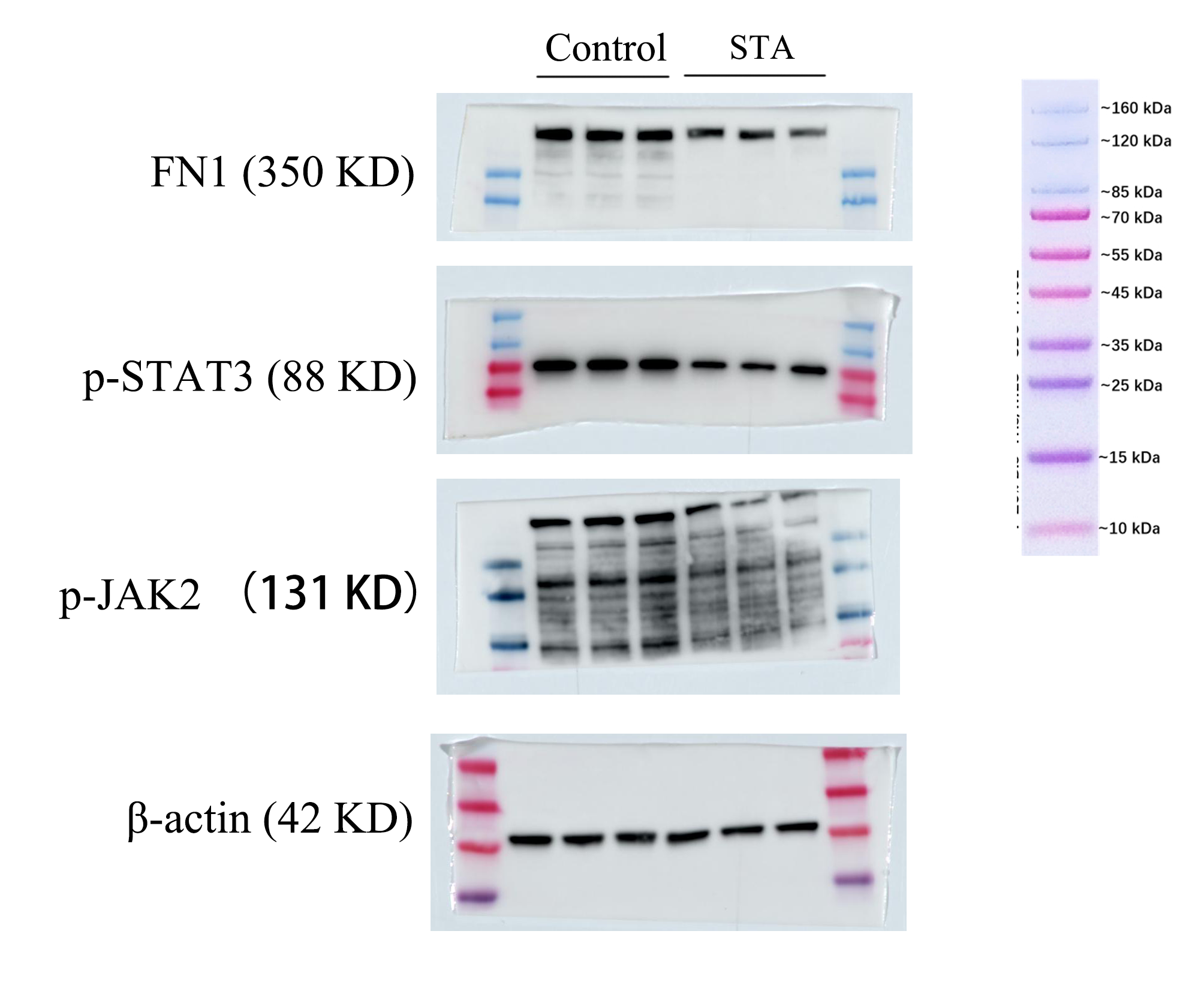

Supplement: Supplementary file 1 [file DataSheet1.docx]
